# East Siberian Arctic inland waters emit mostly contemporary carbon

**DOI:** 10.1038/s41467-020-15511-6

**Published:** 2020-04-02

**Authors:** Joshua F. Dean, Ove H. Meisel, Melanie Martyn Rosco, Luca Belelli Marchesini, Mark H. Garnett, Henk Lenderink, Richard van Logtestijn, Alberto V. Borges, Steven Bouillon, Thibault Lambert, Thomas Röckmann, Trofim Maximov, Roman Petrov, Sergei Karsanaev, Rien Aerts, Jacobus van Huissteden, Jorien E. Vonk, A. Johannes Dolman

**Affiliations:** 1 Department of Earth Sciences, Vrije Universiteit Amsterdam, Amsterdam, the Netherlands; 2 School of Environmental Sciences, University of Liverpool, Liverpool, UK; 3 Department of Sustainable Agro-ecosystems and Bioresources, Research and Innovation Centre, Fondazione Edmund Mach, San Michele all’Adige, Italy; 4 Department of Landscape Design and Sustainable Ecosystems, Agrarian‐Technological Institute, RUDN University, Moscow, Russia; 5 Natural Environment Research Council Radiocarbon Facility, East Kilbride, UK; 6 Department of Ecological Sciences, Vrije Universiteit Amsterdam, Amsterdam, the Netherlands; 7 Chemical Oceanography Unit, University of Liège, Liège, Belgium; 8 Department of Earth and Environmental Science, Katholieke Universiteit Leuven, Leuven, Belgium; 9 Institute for Marine and Atmospheric Research, Utrecht University, Utrecht, the Netherlands; 10 Institute for Biological Problems of the Cryolithozone, Siberian Branch Russian Academy of Sciences, Yakutsk, Russia; 11 North-Eastern Federal University, Yakutsk, Russia

**Keywords:** Carbon cycle, Cryospheric science

## Abstract

Inland waters (rivers, lakes and ponds) are important conduits for the emission of terrestrial carbon in Arctic permafrost landscapes. These emissions are driven by turnover of contemporary terrestrial carbon and additional pre-aged (Holocene and late-Pleistocene) carbon released from thawing permafrost soils, but the magnitude of these source contributions to total inland water carbon fluxes remains unknown. Here we present unique simultaneous radiocarbon age measurements of inland water CO_2_, CH_4_ and dissolved and particulate organic carbon in northeast Siberia during summer. We show that >80% of total inland water carbon was contemporary in age, but pre-aged carbon contributed >50% at sites strongly affected by permafrost thaw. CO_2_ and CH_4_ were younger than dissolved and particulate organic carbon, suggesting emissions were primarily fuelled by contemporary carbon decomposition. Our findings reveal that inland water carbon emissions from permafrost landscapes may be more sensitive to changes in contemporary carbon turnover than the release of pre-aged carbon from thawing permafrost.

## Abstract (web summary)

The release of ancient carbon from thawing permafrost is thought to have an important impact on global biogeochemistry through positive feedbacks. Here Dean and colleagues show that in Siberian permafrost, warming could liberate more contemporary carbon relative to aged counterparts.

## Introduction

Inland waters are highly abundant and important pathways for the export of terrestrial carbon (C) in northern high-latitude permafrost landscapes where ~50% (~1300 Pg C) of the global soil organic C pool is stored^1,2^. This C is increasingly vulnerable to destabilization and release due to permafrost thaw driven by rising Arctic air temperatures^2–4^. Inland waters in stable permafrost landscapes primarily receive terrestrial C from contemporary biological turnover within seasonally thawed topsoils^5–7^. As these landscapes warm, it is likely more contemporary C will be released to inland waters from sustained and enhanced biological turnover and event-based vegetation die-off (Arctic greening versus browning)^8,9^. As permafrost soils thaw, additional old C that had remained frozen since the Holocene is also released to inland waters^7^. Twenty-five percent of permafrost soil C is located in regions with substantial deposits of even older, ancient Pleistocene-aged sediments called Yedoma that is particularly vulnerable to thaw due to its high ice content, the majority of which is located in northeast Siberia^1^. These contemporary terrestrial C and old and ancient (pre-aged) C sources are released to inland waters in four main forms: dissolved and particulate organic C (DOC < 0.2–0.7 µm in size <POC), dissolved inorganic C (including CO_2_), and CH_4_ (ref. ^2^). Shifts in the ratio of these C forms from one to another, or increases in total inland water C concentrations, can drive the magnitude of potential climate feedbacks^2,10,11^.

Terrestrial C can be readily decomposed to CO_2_ and CH_4_ during transport and storage in inland waters and this is thought to be an important driver of permafrost landscape C emissions. Radiocarbon (^14^C) based studies in Yedoma regions have shown that pre-aged C is highly vulnerable to microbial decomposition to CO_2_ when released into streams^12–14^, while studies targeting thermokarst lakes (formed from abrupt thaw processes) detected CH_4_ emissions as old as 42,900 years before present (yBP)^15^. In contrast, contemporary C dominated emissions to the atmosphere in non-Yedoma permafrost regions^6,7,16,17^, and ice-core studies suggest it is unlikely that large-scale emissions of pre-aged CH_4_ from permafrost regions have occurred in the past ~15,000 years^18^. It therefore remains unclear whether the release of pre-aged permafrost C into inland waters is an important driver of landscape-scale C emissions relative to contemporary C turnover.

**Fig. 1:**
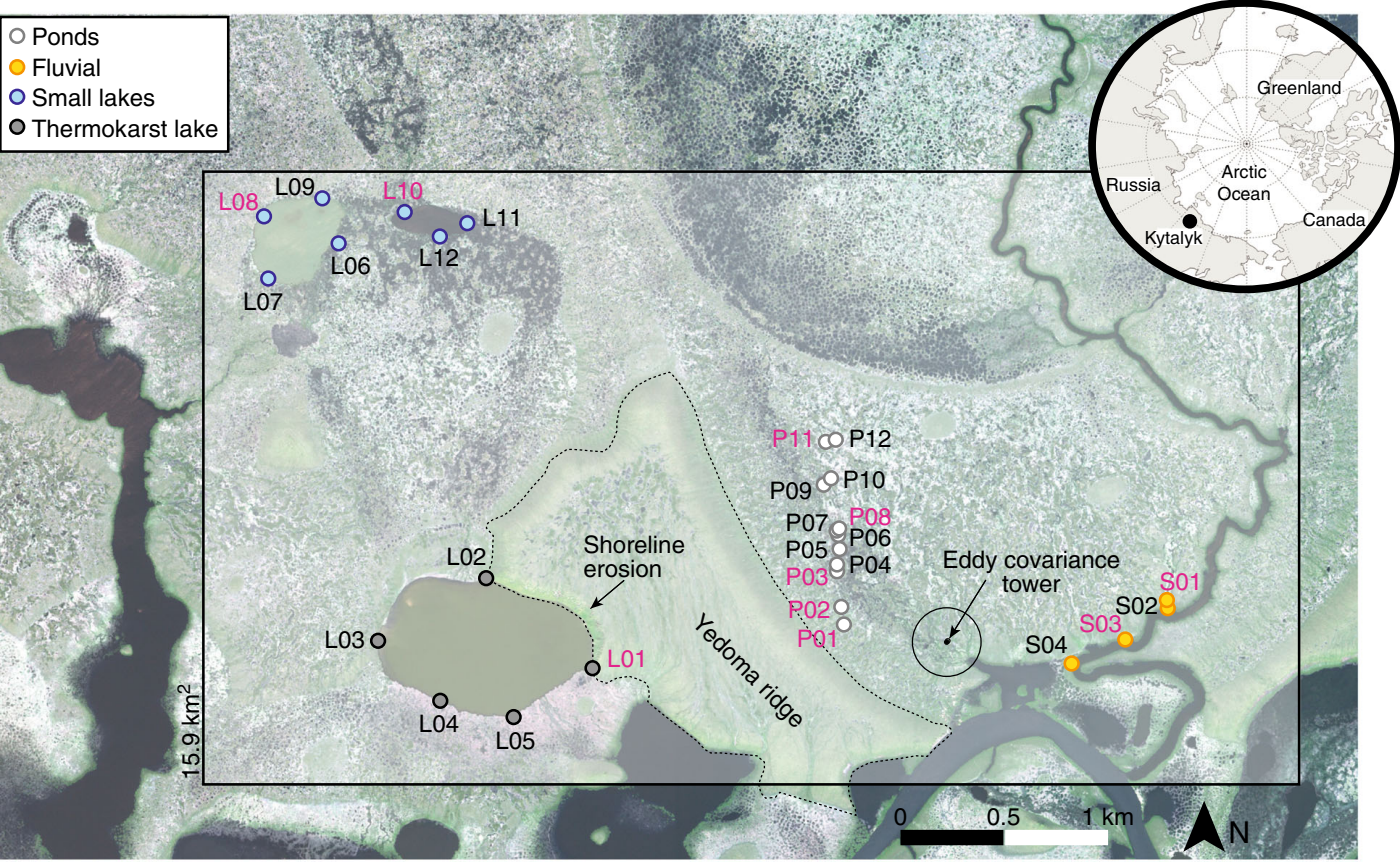
Study region and sampling locations. Sampling locations for concentrations (black text) and radiocarbon (magenta text), eddy covariance tower (black dot) and its footprint (black circle represents 75–90% of flux contributions^44^), Yedoma ridge (dashed black outline), and location of the site within the Arctic (inset). The box (15.9 km^2^) indicates the area used for upscaled fluxes (Table 1). Background is a panchromatic Worldview satellite image from 15 July 2015, WorldView © 2019 MAXAR.

Here we quantify the contributions of pre-aged and contemporary C in all forms to inland waters of a lowland permafrost landscape. Using an isotope mixing model, we determine the age of C contributions to inland water emissions from simultaneous ^14^C and concentration measurements of DOC, POC, and dissolved CO_2_ and CH_4_ across a selection of inland water sites in the continuous permafrost zone of the northeast Siberian tundra (Fig. 1). We focus on dissolved CO_2_ and CH_4_ rather than ebullition (bubble release) because the dissolved gases provide a more spatiotemporally integrated measure of high-latitude inland water greenhouse gas release^19^. The selected sites cover shallow tundra ponds (<0.5 m deep, 3–240 m^2^ in area) with very limited access to pre-aged C (sites P01–12), fluvial systems consisting of an irregularly connected pond and a ~5 m wide stream draining a drained lake basin (sites S01–04), two small lakes (<3 m deep, 0.04 and 0.14 km^2^) in thaw-depression and drained lake basin dominated tundra (sites L06–12), and a thermokarst lake (up to 7 m depth, 0.51 km^2^; sites L01–05) that is eroding into a Yedoma ridge (Fig. 1; Table 1 and Supplementary Table 1). ^14^C samples were also collected from meltwater draining directly from a thawing Yedoma ice mass in the eroding bank of the thermokarst lake (adjacent to site L01 in Fig. 1) to assess the maximum potential age of C mobilized into solution during Yedoma thaw. We expected contemporary C to dominate in the pond, fluvial and small shallow lake sites where inputs of pre-aged C are limited, and pre-aged C to dominate in both the thermokarst lake, which actively erodes into Yedoma sediments, and the Yedoma meltwater. We aimed to capture a snapshot of the likely oldest C released during the growing season by collecting samples in July–August 2016 when the seasonally thawed active layer was deepest and thermokarst erosion was at its maximum (Supplementary Fig. 1)^7,20^. However, it is worth noting that the oldest C emitted to the atmosphere by such inland waters may occur at the end of winter due to the buildup of CO_2_ and CH_4_ under ice, which also isolates inland waters from contemporary inputs^19^. Inland water C emissions were upscaled for the 15.9 km^2^ study region (Fig. 1), and the relative contributions of pre-aged and contemporary C were estimated and contextualized with tundra emissions measured using eddy covariance. The study region is representative of wider northeast Siberian Arctic landscapes, which are dominated by ponds, fluvial networks, drained thaw lake basins and remnant Yedoma deposits^21–23^.

**Table 1:** Carbon concentrations/fluxes, upscaled emissions and sources during the study period (25 July–17 August 2016).

Sample location	DOC (mg L^−1^)	POC (mg L^−1^)	CO_2_ (mg C m^−2^ d^−1^)	CH_4_ (mg C m^−2^ d^−1^)	Coverage of study area	Upscaled emissions to the atmosphere (Mg C)	Proportion derived from contemporary carbon sources	Proportion derived from pre-aged carbon sources
Ponds	25.6 ± 11.8	3.6 ± 3.6	364 ± 250	20 ± 30	4.9%^a^	6.9 ± 5.0	$0.96_{ - 0.21}^{ + 0.04}$	0.04 ± 0.03
Fluvial	2.4–12.1^b^	1.9 ± 1.4	1820 ± 1118	56 ± 29	1.2%	8.2 ± 5.0	$0.84_{ - 0.54}^{ + 0.16}$	0.16 ± 0.10
Small lakes	8.2 ± 1.2	1.4 ± 0.8	394 ± 89	18 ± 6	1.2%	1.8 ± 0.4	$0.83_{ - 0.53}^{ + 0.17}$	0.17 ± 0.10
Thermokarst lake	6.5 ± 1.2	2.4 ± 3.4	290 ± 232	21 ± 7	3.2%	3.6 ± 2.8	0.50 ± 0.40	0.50 ± 0.35
Yedoma meltwater	42.5^c^	8.5^c^	nd	nd	nd	nd	0.47 ± 0.41	0.53 ± 0.40
Tundra	nd	nd	−2784 ± 349	42 ± 5	89.4%	−897.4 ± 115.9	nd	nd

All values are mean ± 1σ, except upscaled fluxes to the atmosphere where values are mean ± range of possible fluxes using CO_2_ and CH_4_ flux 1σ uncertainties; proportional contributions of contemporary and pre-aged sources are those calculated for all C forms (Supplementary Table 2); proportional contributions for CO_2_ and CH_4_ only, which are used in the flux upscaling (see Results), are reported in Supplementary Table 2.

nd not determined.

^a^From ref. ^23^.

^b^*n* = 2, ^c^*n* = 1, values taken from radiocarbon samples.

^c^*n* = 1.

## Results

### Inland water carbon isotope compositions

**Fig. 2:**
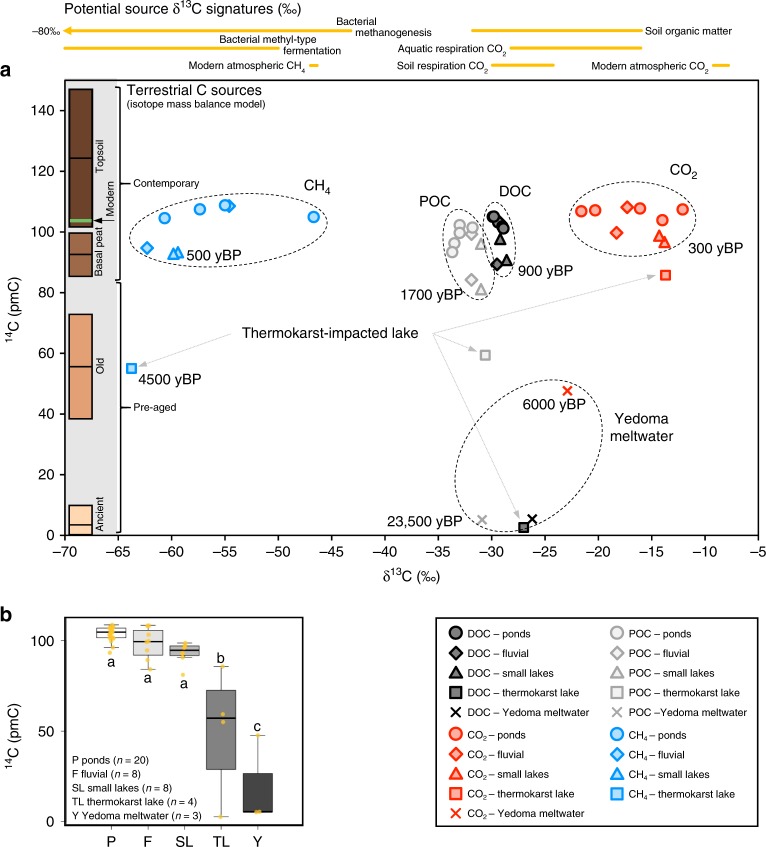
Isotope (^14^C and δ^13^C) composition of all carbon forms. **a** Dissolved organic carbon (DOC), particulate organic carbon (POC), and dissolved carbon dioxide (CO_2_) and methane (CH_4_) across all sample locations; the bars on the left indicate the ^14^C (not δ^13^C) values (mean ± 1σ) of the five sources used in the isotope mass balance; ^14^C ages are indicative (years before present; yBP); δ^13^C signatures of potential carbon sources are indicated at the top: modern atmospheric CH_4_ (ref. ^45^), bacterial methanogensis and bacterial methyl-type fermentation^24^, aquatic (permafrost freshwaters) respiration CO_2_ (refs. ^19,26^), soil respiration CO_2_ and soil organic matter from the study site (including Yedoma soils)^22^, and modern atmospheric CO_2_ (ref. ^25^). **b** ^14^C signatures across all carbon forms by sample location; the thick horizontal lines represent the median, limits of the boxes represent upper and lower quartiles, whiskers extend to 1.5 times the interquartile range, dots represent all data points, letters indicate statistical differences between the sample locations using ANOVA (see Methods).

The ^14^C ages across the different sampling locations revealed an age gradient ranging from modern (post-1950 CE) to ancient (29,355 ± 2967 yBP; mean ± 1σ; Fig. 2a): pond sites were the youngest, followed by increasing ^14^C ages in the fluvial, small lake, thermokarst lake and Yedoma meltwater sites (Fig. 2b). Pond, fluvial and small lake ^14^C ages were statistically alike, ranging from 1679 ± 38 yBP to modern; thermokarst lake and Yedoma meltwater ^14^C ages were significantly older (29,355 ± 2967 to 1234 ± 38 yBP; Fig. 2b).

^14^CO_2_ values were consistently younger than the other ^14^C forms and ^14^CH_4_ was generally younger than PO^14^C, based on linear regressions testing for age divergence between the different ^14^C forms. These regressions were sensitive to extreme values due to limited sample numbers, so thermokarst lake and Yedoma ^14^C values were excluded initially. All ^14^C forms were significantly correlated with one another (*p* < 0.05; *R*^2^ = 0.54–0.94; Supplementary Fig. 2). Including the thermokarst and Yedoma samples increased the level of correlation (*p* < 0.001; *R*^*2*^ = 0.74–0.98; Supplementary Fig. 3), but CO_2_ and CH_4_ were still younger than DOC and POC (except thermokarst lake CH_4_ and POC, which were approximately the same; Fig. 2a).

The δ^13^C–CO_2_ values reflect mixing of respiration of recent and decomposed organic matter with some contribution from methanogenesis as well^24,25^. The less negative δ^13^C–CO_2_ values (−22.9 to −12.1‰) in comparison with CH_4_ were consistent with CO_2_ samples collected from Canadian Arctic inland waters^7^. The least negative δ^13^C–CO_2_ values indicated there could be some exchange with atmospheric CO_2_ which has a less negative δ^13^C value (−9‰) than respired organic matter (−30.0 to −16.2‰)^19,22,26^, but all sites were supersaturated with CO_2_ compared with the atmosphere, limiting ingress of atmospheric CO_2_ into the water. The δ^13^C–CH_4_ values indicated production from microbial methanogenesis (−63.8 to −46.7‰), although the least negative value was close to thermogenic CH_4_ (−45 to −20‰)^24^ but this could also reflect enrichment by partial methane oxidation^19,24^. The isotopic separation factor (*ε*_C_) of δ^13^C–CO_2_ and δ^13^C–CH_4_ (≈δ^13^C–CO_2_ − δ^13^C–CH_4_) can indicate the pathway of CH_4_ production. The *ε*_C_ values in this study were 32.7–45.6 (42.1 ± 4.8 [median ± 1σ]; Supplementary Data 1), which indicates CH_4_ formation from bacterial methyl-type fermentation (decomposition of methylated organic matter compounds; Fig. 2a) as opposed to the reduction of carbonate, thermogenic sources, or exchange with the atmosphere^24^. Geological contributions to CO_2_ or CH_4_ isotopic signatures are very unlikely at the study site (see Methods). The δ^13^C-DOC values (−29.9 to −26.2‰) were consistent with freshwater DOC derived from the C3 photosynthetic pathway^7,25,27^; δ^13^C-POC values were more negative (−33.7 to 30.6‰), reflecting the presence of vegetation fragments^25^ (Fig. 2a).

### Contemporary versus pre-aged carbon sources

**Fig. 3:**
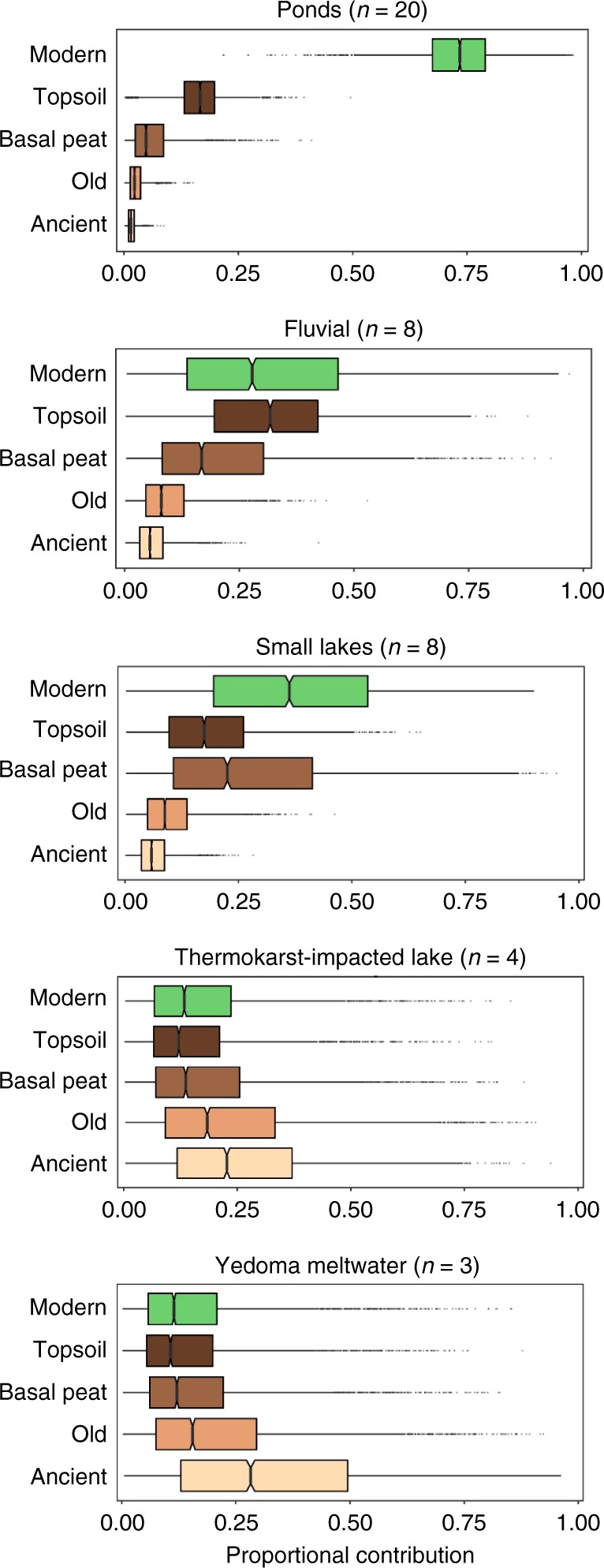
Relative source contributions to total inland water carbon. Estimated from a five-source Bayesian isotope mass balance model: modern (2013) atmospheric carbon dioxide (CO_2_); recent, 1950–2012 carbon fixed into plant and soil organic matter in the topsoil; basal peat ages measured at the site representing the oldest carbon available from Holocene peat accumulation (250–1180 yBP)); old carbon in late-Pleistocene to early Holocene soils (1765–9970 yBP); and ancient Pleistocene-aged Yedoma carbon (>27,920 yBP; Fig. 2a)^6,7,22^. Notched lines in the boxes represent the median, limits represent upper and lower quartiles, whiskers extend to 1.5 times the interquartile range, dots represent data points beyond this range.

A five-source isotope mass balance model^6,28,29^ was used to estimate relative contributions to the measured ^14^C values from C sources measured at the study site^22^. These C sources were defined as contemporary (modern atmospheric C, topsoil and late-Holocene basal peat: 2013 CE to 1180 yBP) and pre-aged C (old Holocene C and ancient Yedoma C: 1765 yBP to >27,920 yBP) (Fig. 2a; see Methods). The majority of inland water C observed in this study was contemporary, but pre-aged C contributed to all inland water C forms where permafrost thaw occurred (Fig. 2a). Pond, fluvial and small lake C was primarily derived from modern and topsoil C sources (>56% ± 33% [mean ± σ]; Fig. 3; Supplementary Table 2). When basal peat sources were included, these three contemporary sources accounted for >$83_{ - 53}^{ + 17}$% of the C in these waters (Table 1). In the thermokarst lake and Yedoma meltwater, the majority of C was pre-aged (>50% ± 35%) (Fig. 3; Table 1).

### Carbon concentrations

**Fig. 4:**
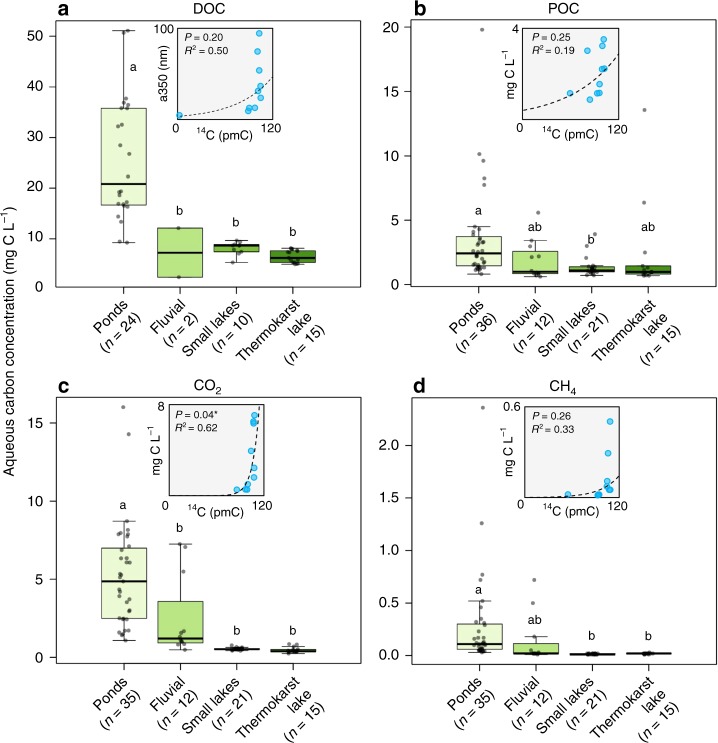
Concentrations of all carbon forms. **a** Dissolved organic carbon (DOC), **b** particulate organic carbon (POC), **c** carbon dioxide (CO_2_), and **d** methane (CH_4_) for each sample location; the thick horizontal lines represent the median, limits of the boxes represent upper and lower quartiles, whiskers extend to 1.5 times the interquartile range, dots represent all data points, letters indicate statistical differences between the sample locations using ANOVA (see Methods). Inset: relationship between ^14^C content and carbon concentrations (absorbance at 350 nm [a350] for DOC; Supplementary Fig. 4)^46^, excluding Yedoma meltwater (see Methods) (*indicates *P* < 0.05).

DOC had the highest concentrations of the four C forms measured (15.8 ± 12.3 mg C L^−1^ [mean ± 1σ]; Fig. 4; Supplementary Fig. 4). POC and CO_2_ concentrations were statistically alike (2.6 ± 3.0 mg C L^−1^ versus 2.8 ± 3.2 mg C L^−1^), lower than DOC but higher than CH_4_ (0.1 ± 0.3 mg C L^−1^). Pond C concentrations were consistently higher than fluvial, small lake or thermokarst lake sites, with the latter three containing statistically alike concentrations (Fig. 4). Yedoma meltwater had the highest organic C concentrations (DOC = 42.5 mg C L^−1^, POC = 8.5 mg C L^−1^; Table 1). Sites with higher CO_2_ concentrations had younger ^14^CO_2_ ages (*p* < 0.05, *R*^2^ = 0.71; the best fit was exponential; Fig. 4c). While this was the only statistically significant relationship, waters with higher overall C concentrations tended to be younger (Fig. 4).

### Dissolved organic matter characteristics

We used absorbance and fluorescence spectroscopy to determine the source and decomposition pathways of inland water dissolved organic matter (DOM) which includes DOC, the C form with the highest concentrations. DOM can be produced by terrestrial and microbial production or decomposed by microbial degradation and photo-oxidation^30^.

The DOM was primarily derived from terrestrial material, and both microbial activity and photo-oxidation played a role in its decomposition. Relatively high humic content (Humification Index [HIX] values = 0.6–0.7), indicates a predominantly terrestrial origin, as do substantial contributions of parallel factor analysis (PARAFAC) components C1, C3, and C4 to the total DOM fluorescence signal, which are associated with high aromaticity and intermediate/high-molecular weight terrestrial DOM (Supplementary Fig. 5). Higher microbial contributions to the DOM pool were associated with older DOC, indicated by increasing biological index (BIX) values and decreasing humic content with older ages (Supplementary Fig. 6). This suggests increased microbial activity that could include the decomposition of organic C to CO_2_ and CH_4_, and/or the incorporation of organic C into microbial biomass. Photo-oxidation likely contributed to the decomposition of younger DOM which had higher molecular weight and aromaticity—characteristic of photosensitive molecules^31^—than older DOM. This trend in molecular weight is shown by an increase in S_R_ (slope ratio) and E_2_:E_3_ ratio with older ages, both inversely proportional to molecular weight, alongside a decrease in percent C1 (proportional contribution of PARAFAC component C1 to the total fluorescence signal) which is associated with high aromaticity and high-molecular weight terrestrial DOM (Supplementary Fig. 6).

### Landscape-scale carbon emissions

The study region was a C sink with a net flux of −876.9 ± 136.4 Mg C during the study period, 25 July–17 August 2016 (summing the upscaled fluxes to the atmosphere in Table 1). This was mainly due to the tundra surrounding the inland waters which was a CO_2_ sink (−2784 ± 349 mg C–CO_2_ m^−2^ d^−1^) partially offset by tundra CH_4_ emissions (43 ± 5 mg C–CH_4_ m^−2^ d^−1^; Table 1). 89.4% of the landscape was tundra (14.2 km^2^; Fig. 1), and thus dominated total C flux per unit area and by spatial extent. Of the inland waters, fluvial sites had the highest CO_2_ emissions (1820 ± 1118 mg C–CO_2_ m^−2^ d^−1^ [mean ± 1σ]; Table 1; Supplementary Fig. 7). Emissions from the pond, small lake and thermokarst lake were all lower than fluvial emissions but not significantly different from one another (290 ± 232 to 394 ± 89 mg C–-CO_2_ m^−2^ d^−1^). The same pattern was evident for CH_4_: fluvial diffusive emissions were 56 ± 29 mg C–CH_4_ m^−2^ d^−1^, compared with 18 ± 6 to 20 ± 30 mg C–CH_4_ m^−2^ d^−1^ from the other sites (Supplementary Fig. 7). CO_2_ and CH_4_ emissions from the fluvial, small lake, and pond fluxes (1.45 km^2^, 7.3% of the study region) were 16.8 ± 10.4 Mg C; the thermokarst lake emissions (0.51 km^2^, 3.2% of the study region) were 3.6 ± 2.8 Mg C. Of these fluxes, contemporary C emissions were 17.0 ± 10.9 Mg C and pre-aged C emissions were 3.5 ± 2.3 Mg C, offsetting 1.9 ± 1.2% and 0.4 ± 0.3% of the tundra sink, respectively.

## Discussion

We demonstrate that the inland waters in this permafrost landscape were primarily fueled by contemporary C. We also present direct evidence that pre-aged C can be simultaneously released in all four main inland water C forms where permafrost thaw is occurring. CO_2_ and CH_4_ tended to be younger than DOC and POC, indicating that contemporary C fuels summer inland water emissions in the study region. This is due to the relatively high abundance of contemporary organic C relative to pre-aged C, and contemporary DOC being more readily photo-oxidized than pre-aged C. The flipside of this finding is that pre-aged inland water C emissions described in previous studies may be caused by the absence of contemporary C as much as by the vulnerability of pre-aged C to microbial decomposition. Given the predominance of labile contemporary C in the inland waters observed in this study, these systems show a high connectivity with contemporary terrestrial C, and are thus likely more sensitive to shifts in contemporary C cycling than the cycling of thawed pre-aged C.

Evidence for contemporary C fueling inland waters in this permafrost landscape is demonstrated by the pond, fluvial and small lake sites having the largest upscaled inland water C emissions (equivalent to 2.3 ± 1.5% of the tundra C sink), of which >80% was contemporary C (Table 1). In general, sites with higher C concentrations tended to be younger (Fig. 4). However, the high proportion of pre-aged C contributing to the thermokarst lake and Yedoma meltwater demonstrates that permafrost thaw will release old and ancient C to all inland water C forms. Across this gradient of inland water ^14^C ages (Fig. 2b), the generally close relationship of CH_4_ with DOC and POC with DOC suggests that C release across these different forms was consistently from similar sources. The divergent relationship between DO^14^C/PO^14^C and ^14^CO_2_, and PO^14^C and ^14^CH_4_ was driven by the younger age of the gaseous C (Fig. 2a). This implies that the C fueling inland water CO_2_ and CH_4_ emissions was generally younger than mobilized DOC and POC^7,32^, and that the most labile organic matter pools were also the youngest.

Permafrost thaw can result in high concentrations of low molecular weight DOC and high POC concentrations being released to inland waters^2,12^. Incubation-based ^14^C studies have suggested that this pre-aged C may be rapidly decomposed to CO_2_ (refs. ^12–14^). Our simultaneous in situ ^14^CO_2_ and ^14^CH_4_ observations were generally younger than DOC and POC suggesting that contemporary C is also being rapidly decomposed in thaw affected inland waters, potentially at a faster rate or in greater proportion than pre-aged C. The utilization of pre-aged C by microbes previously demonstrated in streams draining thawing ancient Yedoma sediments^12–14^ could therefore just be due to the limited availability of contemporary organic C in those systems. This effect is amplified by these studies targeting streams heavily impacted by thermokarst activity, and the incubation approaches used which restrict contemporary inputs entirely. Our findings show that the large amount and high lability of contemporary C mean that it can dominate C cycling compared with thawed pre-aged C in permafrost systems at the landscape-scale.

The majority of terrestrial C released into the study inland waters was in the form of DOC (Fig. 4)^33^. Our DOM spectroscopy results indicated that the decomposition pathways for DOC was dependent on its source: younger DOC was more vulnerable to photo-oxidation whilst older DOC was more vulnerable to microbial decomposition. Pre-aged permafrost DOC is known to be readily decomposed by microbes^12–14^, and this is supported by the older CO_2_ and CH_4_ ages observed in our thermokarst lake and Yedoma meltwater (Fig. 2a). Photo-oxidation can also actively decompose DOC in Arctic inland waters^34^, but is less effective at decomposing ancient Yedoma C^35^. Our samples were collected in late summer when daylight hours can extend for virtually the whole day, so photo-oxidation was likely an important process of organic C decomposition. This is supported by the predominance of contemporary CO_2_ and CH_4_, indicating that photo-oxidation of contemporary organic C may be a proportionally more important contributor to inland water emissions in the summer months than microbial decomposition, depending on how much CO_2_ and CH_4_ is sourced from organic C decomposition in the surrounding soils.

Decomposition of pre-aged organic C has most notably been demonstrated in CH_4_ ebullition from thermokarst lake sediments that was up to 42,900 yBP in age^15^. In the present study, dissolved CO_2_ emissions were larger and younger than dissolved CH_4_, consistent with previously observed Arctic inland water emissions (3–6420 mg C–CO_2_ m^−2^ d^−1^ versus 1.0–182 mg C–CH_4_ m^−2^ d^−1^)^6,7,36–40^. Both dissolved CO_2_ and CH_4_ emissions were younger and tended to be smaller than CH_4_ ebullition (0–255 mg C–CH_4_  m^−2^ d^−1^)^40^ which was not measured in our study. CH_4_ ebullition is usually generated at depth, as opposed to dissolved CH_4_ which represents a spatiotemporally integrated signal of lake sediment and terrestrial inputs^19^. The generally older age of CH_4_ ebullition compared with dissolved CH_4_ indicates that where contemporary organic C is limited, such as deep unfrozen lake sediments (taliks), pre-aged C will drive CH_4_ production^15^. For example, in Alaskan lakes, dissolved CO_2_ and CH_4_ collected in winter were older than in summer. This was because frozen lake surfaces prevented inputs of contemporary terrestrial C and stopped ebullition reaching the surface, which allow these bubbles containing old CH_4_ and CO_2_ to dissolve into the water column (refs. ^6,19^). These age differences further indicate that microbial utilization of pre-aged C is due to the restricted supply of contemporary organic C, rather than preferential mineralization of pre-aged organic matter.

Inland waters can rapidly turnover contemporary organic C^32,41^, and the few existing in situ ^14^C measurements of dissolved CO_2_ and CH_4_ across the Arctic have been contemporary in age^6,7^. The export of pre-aged C by inland waters from thawing permafrost landscapes may instead occur as DOC and POC. Our comparison of inland water CO_2_ and CH_4_ emissions with the measured terrestrial sink does not account for lateral transport of organic C. With DOC and POC concentrations consistently *c*. 4 times higher and generally older than diffuse CO_2_ and CH_4_ (Figs. 2 and 4), lateral transport is a potentially important component of landscape C loss in the region. Mobilized DOC and POC may be transported laterally to lake, fluvial or marine systems where it can be decomposed to CO_2_ and CH_4_, or buried in sedimentary reservoirs^2,42^. Rates of lateral transport may be greater at other times of the year than our study period depending on hydrological regimes, e.g., during the Spring snow-melt pulse, and as a result of abrupt thaw events^2^. It is therefore important to track all C forms across different times of the year to fully capture the export of pre-aged permafrost C by inland waters.

Our study region had a slightly lower proportional coverage of inland water bodies compared with a selection of Arctic regions in a recent study: of 31 sites, 29 had higher proportional inland water coverage but only by 4.5 ± 3.9% (mean ± 1σ)^23^. Inland waters types and their areal coverage in permafrost landscapes could increase or decrease under climate change depending on localized topography and drainage patterns^4,21,23,43^. There is thus a need to capture C dynamics across a range of water types within the same landscapes, as shown by the variable C concentrations and isotopic compositions seen across our study sites.

Our observations demonstrate that inland waters in permafrost landscapes are sensitive to both contemporary and pre-aged C release, although contemporary C dominates in this landscape. This means that regardless of whether the changing Arctic climate will increase inputs of contemporary or pre-aged C to inland waters, their annual CO_2_ and CH_4_ emissions will likely go up, offsetting terrestrial C sinks. In the study region, inland water CO_2_ and CH_4_ emissions were a small offset of the terrestrial sink (2.3 ± 1.5% in total), so large increases in inland water coverage and supply of terrestrial C to these waters is needed in order for them to significantly offset the terrestrial sink.

## Methods

### Study approach

The study site is in the oligotrophic tundra of the Kytalyk Nature Reserve in the Indigirka River lowlands^22^ (70.83°N, 147.49°E; Fig. 1). Average active layer depths in the tundra around and beneath the ponds during the study period, July–August, were 26 ± 12 cm (mean ± 1σ, *n* = 370; Table S1). The landscape is dominated by a series of drained lake basins formed by thaw driven subsidence and drainage^21^, and the floodplain of the Berelekh River, a major tributary to the Indigirka. A single Yedoma ridge (up to 30-m high) lies within the study region, into which the thermokarst lake is eroding and from which the Yedoma meltwater samples were collected. This ridge is an remnant of organic-rich, ~2–6% C by mass, Pleistocene-aged permafrost sediments deposited as loess or fluvial silts with ice content 30–90% by volume^22^. The ridge covers ~12% of the landscape surface and is potentially distributed throughout the region as a result of thaw and erosion^22^ (Fig. 1); such deposits are common throughout northeast Siberia^1^.

The study region is underlain by continuous permafrost limiting potential geological inputs to aquatic CO_2_ or CH_4_, and neither ^14^CO_2_ nor δ^13^C–CO_2_ (which are in isotopic equilibrium with total dissolved inorganic C) correlated with geochemical indicators of rock weathering (e.g., water Ca^2+^ concentrations). Geological inputs, such as rock weathering^47^ or CH_4_ seeps^48^, were therefore unlikely to have influenced our observations.

Mean air temperatures during the study period (25 July–17 August 2016) were 10.3 ± 3.3 °C, 7.6 ± 4.7 °C during the growing season (June–September 2016), and −12.4 ± 16.7 °C in the year preceding the sampling campaign. These values are within normal temperature ranges for the site (monthly averages range from 9.7 °C in June to −34.2 °C in January)^22^. Precipitation at the study site occurs mainly as rain during the summer, with an annual mean of 232 mm^22^; during the sampling period, 30 mm of rain fell compared with 129 mm over the 2016 growing season (Supplementary Fig. 1). Vegetation in the tundra areas of the site consists primarily of *Eriophorum vaginatum* and *Eriophorum augustifolium*, *Betula nana*, *Carex* sp. and *Sphagnum* sp. The ponds tend to be surrounded by *E*. *vaginatum*, *E*. *augustifolium*, *B*. *nana*, *Carex* sp., and *Sphagnum* sp., while the fluvial systems are bordered primarily by *Arctophila fulva*, *Arctagrostis latifolia*, and *Salix pulchra*^49^.

### Sampling and analyses

We collected concurrent ^14^C, δ^13^C, and C concentration samples for DOC, POC, and dissolved CO_2_ and CH_4_ from fluvial, pond, small lake and thermokarst lake sites, and Yedoma meltwater. Samples were collected from the edges of water bodies as could be accessed from the shoreline. Multiple C concentration samples were collected from the lake and stream locations to account for spatial variability. Only one location was sampled for ^14^C and δ^13^C in each of the study lakes due to the prohibitive cost of analyses. However, ^14^C in dissolved CO_2_ and CH_4_ have been shown to be relatively uniform in lakes of equivalent size in Alaska^19^. The stream was sampled in two locations and the five different ponds were sampled for ^14^C and δ^13^C, but again widespread sampling was limited due to cost. Ponds were only sampled once since their small size means that variations in C source are likely small (Table S1). DOC and POC, and dissolved CO_2_ and CH_4_ concentration samples were collected three times from each site during the study period (except Yedoma meltwater, where *n* = 1) at three-day intervals in daylight hours for consistency. DOM samples were also collected at the same frequency as the concentration samples but only at sites where ^14^C samples were collected (Fig. 1; except Yedoma meltwater, where *n* = 1). ^14^C and δ^13^C samples were collected at a single time point from a selection of these sites between 05 and 09 August 2016 (Fig. 1; Supplementary Data 1)^50–54^; additional δ^13^C samples were also collected for CH_4_ and DOC for quality control^55–57^ (see Supplementary Methods; Supplementary Fig. 8; Supplementary Data 1 and 2).

### Isotope mass balance model

The Stable Isotope Mixing Models in R (simmr) package^28^ was used to estimate relative contributions of potential C sources to ^14^C samples from each group of sample sites (fluvial, pond etc.; Supplementary Table 2). The isotope mass balance model was only used for ^14^C because δ^13^C is fractionated during CO_2_ and CH_4_ production, and so a dual isotope mass balance, common in aquatic organic matter studies^58^, would not work. This approach therefore provides the theoretical possible contributions of different sources following previous studies^6,29^, but here the sources are further constrained by using measured soil organic matter ^14^C values obtained from the study region^22^. While some water bodies, for example the ponds (see Introduction), are unlikely to receive contributions from pre-aged C sources, we applied the isotope mass balance model with the same end-members to all samples for consistency to demonstrate all possible source contributions that could produce the observed ^14^C values. We used simmr to carry out a mass balance (Eq. 1), fitting the potential contributions of five different C sources (see below) using Markov chain Monte Carlo modeling. Ranges and uncertainties in the ^14^C signature of the different sources were propagated through the model, and model fitting carried out by the *Just Another Gibbs Sampler* code. Model convergence was only considered acceptable when the upper confidence interval of the final model outputs was 1.00 ± 0.10 (ref. ^28^). We excluded any food web specific functions from the mass balance, such as trophic enrichment factors, or preferential source contributions^28^.

1 $$C14_{{\mathrm{group}}} = C14_{{\mathrm{modern}}} + C14_{{\mathrm{topsoil}}} + C14_{{\mathrm{basal}}\,{\mathrm{peat}}} + C14_{{\mathrm{old}}} + C14_{{\mathrm{ancient}}}$$

*C*14_group_ represents the ^14^C content of the different inland water C components within each group of sample sites (in pmC; Supplementary Data 1), which is the sum of the mass balance between the proportional contributions of five different C sources; to achieve mass balance, the relative contributions of the five different sources is assumed to sum to 1. These five sources follow the model used by ref. ^6^ but is adapted here to incorporate soil/sedimentary ^14^C data obtained from the current study site^22^ (Fig. 3). The modern CO_2_ source (*C*14_modern_) was taken as the most recently available atmospheric ^14^CO_2_ value: calendar year 2013 from Barrow, Alaska (103.13 pmC)^6^, only 3-years earlier than the sampling year in the present study. The topsoil source (*C*14_topsoil_) represents the uppermost soil C accumulation in the peat soils, reflecting atmospheric ^14^CO_2_ signatures between 1950 and 2012 (123.64 ± 22.61 pmC [mean ± 1σ])^7^. Basal peat ages (*C*14_basal peat_) represent the oldest C available from the Holocene peat accumulation layers on top of the Yedoma soils (91.95 ± 7.16 pmC)^22^. The old C source (*C*14_old_) represents deep layers of early Holocene to late-Pleistocene C that has come to the surface via cryoturbation (55.13 ± 17.19 pmC)^22^. Ancient C (*C*14_ancient_) represents Pleistocene-aged Yedoma C that has been exposed by thermokarst activity and erosion (3.09 ± 6.19 pmC)^22^.

### Inland water CO_2_ and CH_4_ emissions

Inland water CO_2_ and CH_4_ fluxes (*F*) were calculated using Eq. (2):

2 $$F = k\Delta C$$

where Δ*C* is the difference between the observed gas concentration in the water and the expected concentration if the dissolved gas was in equilibrium with the atmosphere, and *k* is the gas transfer velocity from water to the atmosphere^59^.

Atmospheric CO_2_ and CH_4_ concentrations were taken from the ambient air samples collected daily during the normal headspace sampling and supplemented with atmospheric concentration data from the eddy covariance tower. Lake *k* values were calculated using the relationship with wind speed at 10 m (*U*_10_ in m s^−1^) and *k*_600_ (*k* normalized to a Schmidt value of 600) from ref. ^60^:

3 $$k_{600} = 2.07 + 0.215 \cdot U_{10}^{1.7}$$

*U*_10_ values were available from the eddy covariance site at 30 min intervals^49^. Pond *k*_600_ values were taken from ref. ^61^ (0.36 cm h^−1^), a low *k*_600_ value in the context of inland water *k*_600_ values. Following ref. ^59^, for fluvial sites we used the boreal and Arctic stream *k*_600_ value from ref. ^62^ (13.1 cm h^−1^), which is at the lower end of fluvial *k* values, but at the high end of inland water *k* values in general. The *k*_600_ values can then be converted to *k*:

4 $$k = \frac{{k_{600}}}{{\left( {\frac{{600}}{{Sc}}} \right)^{ - 0.5}}}$$

where *Sc* is the Schmidt number in freshwater computed from water temperature^59^.

### Tundra CO_2_ and CH_4_ emissions

Tundra fluxes at the study site were calculated using the eddy covariance method^63^. The eddy covariance tower at the study site (Fig. 1) was installed in 2003 to measure net ecosystem exchange, using an ultrasonic anemometer (Gill Instruments, Lymington, UK, type R3-50) for wind speed and temperature, and water vapor and CO_2_ concentrations were measured by an open-path infra-red gas analyzer (LI-COR LI-7500, Lincoln, NE, USA)^49^. An open-path infra-red gas analyzer (LI-COR LI-7700) has been used to measure atmospheric CH_4_ concentrations since 2014. All sensors measure at 10 Hz and are installed at a height of 4.7 m. Energy balance closure and good co-spectral shapes required for validation of the eddy covariance methods were demonstrated at the site previously^44,49^.

Tundra fluxes were calculated via EddyPro (version 6.2.2) to provide 30 min mean fluxes. Processing configuration included: 2D rotation of sonic anemometer coordinates, compensation of air density fluctuations (WPL correction), spectroscopic corrections (for LI7700 – CH_4_ flux only) and spectral corrections. Fluxes of CO_2_ and CH_4_ associated with implausible values of gas concentrations, wind speed or sonic temperature caused by frost, precipitation, water condensation on the analyzer lenses or other conditions of sensors disturbance were removed. CO_2_ fluxes were additionally despiked following standard procedure^64^. Daily fluxes (μmol CO_2_ m^−2^ s^−1^ and nmol CH_4_ m^−2^ s^−1^) were summed and daily standard deviations propagated (by calculating the square root of the sum of squares of daily 1σ uncertainties) for the 23-day study period (25 July–17 August 2016), then converted to a daily C flux (mg C m^−2^ d^−1^ ± 1σ; Table 1). Values reported in Table 1 are comparable with CO_2_ fluxes from the same time period in 2003 to 2013, ranging from −1.4 to −2.3 g C m^−2^ d^−1^, and CH_4_ fluxes from the same time period in 2008 to 2015 (pre-2014 fluxes are from chamber measurements at the site)^49^, ranging from 14.5 to 21.0 g C m^−2^ d^−1^. These values are in good agreement with year round fluxes from the site^44,49^ (Supplementary Fig. 1).

### Upscaling emissions to the study region

Daily fluxes measured during the 23-day study period were upscaled to a 15.9-km^2^ study area that encompassed all the study sites (Fig. 1). The area selected for upscaling captures all the landscape to which we know our measurements apply, i.e., all the lakes, ponds and the stream we sampled plus the surrounding tundra, but we did not try and upscale beyond this. The footprint of the eddy covariance tower captures an area of tundra that reflects the majority of land surrounding the sampled inland water locations, and is considered to be representative of the tundra CO_2_ and CH_4_ cycling in the immediate region^44^ (Fig. 1). Areal coverage of the fluvial system (0.2 km^2^), the small lakes (0.2 km^2^) and the thermokarst lake (0.5 km^2^) were calculated from Worldview satellite imagery on 15 July 2015 (Fig. 1). Pond coverage was obtained at the study site from satellite imagery and aerial photography from 2002 to 2013 site in a recent study using image classification (4.9 ± 0.2% of the study region)^23^. Daily fluxes of grouped sample locations (fluvial, pond etc.) were multiplied by 23 days and the relative areas of each inland water system to give a total flux to the atmosphere in Mg C for the period 25 July–17 August 2016. 1σ uncertainties in the flux estimates were propagated through the upscaling by providing maximum and minimum values in the total upscaled fluxes (*x* ± *y* in Table 1). Relative contributions to fluxes from contemporary (atmospheric, topsoil and basal peat C) versus pre-aged (old and ancient C) sources were estimated by multiplying the fluxes by the proportional contributions of these sources to the total C load (Supplementary Table 2).

### Statistics

Statistics were analyzed in R (version 3.5.1). Linear models were used to analyze correlations between groups, and the strength of relationships assessed using analysis of variance (ANOVA), with post hoc Tukey’s honestly significant difference tests (significance level: *p* < 0.05) carried out using the *agricolae* package for letters denoting group similarity in, e.g., Fig. 4. Correlations between variables were assessed using Pearson’s correlations in the *Hmisc* package (e.g., *p*-values in Fig. 4). Yedoma meltwater values were excluded from the correlation with C concentrations in Fig. 4 (insets) because they represent a Yedoma signal that has yet to be released into an inland water system (unlike the thermokarst lake samples), and because there was no Yedoma ^14^CH_4_ value, nor CO_2_ or CH_4_ concentration values, to allow a complete comparison of all correlations.

## Supplementary information

Supplementary Information

Peer Review File

Description of Additional Supplementary Files

Supplementary Data 1

Supplementary Data 2

## Data Availability

All data is available in the Supplementary information and Supplementary Data 1 and 2.

## References

[1] Strauss J (2017). Deep Yedoma permafrost: a synthesis of depositional characteristics and carbon vulnerability. Earth-Sci. Rev..

[2] Vonk JE (2015). Reviews and syntheses: effects of permafrost thaw on Arctic aquatic ecosystems. Biogeosciences.

[3] Box JE (2019). Key indicators of Arctic climate change: 1971–2017. Environ. Res. Lett..

[4] Turetsky MR (2019). Permafrost collapse is accelerating carbon release. Nature.

[5] Raymond PA (2007). Flux and age of dissolved organic carbon exported to the Arctic Ocean: a carbon isotopic study of the five largest arctic rivers. Glob. Biogeochem. Cycles.

[6] Elder CD (2018). Greenhouse gas emissions from diverse Arctic Alaskan lakes are dominated by young carbon. Nat. Clim. Change.

[7] Dean JF (2018). Abundant pre-industrial carbon detected in Canadian Arctic headwaters: implications for the permafrost carbon feedback. Environ. Res. Lett..

[8] Phoenix GK, Bjerke JW (2016). Arctic browning: extreme events and trends reversing arctic greening. Glob. Chang. Biol..

[9] Xu L (2013). Temperature and vegetation seasonality diminishment over northern lands. Nat. Clim. Change.

[10] Schuur EAG (2015). Climate change and the permafrost carbon feedback. Nature.

[11] Dean JF (2018). Methane feedbacks to the global climate system in a warmer world. Rev. Geophys..

[12] Mann PJ (2015). Utilization of ancient permafrost carbon in headwaters of Arctic fluvial networks. Nat. Commun..

[13] Drake TW, Wickland KP, Spencer RGM, McKnight DM, Striegl RG (2015). Ancient low–molecular-weight organic acids in permafrost fuel rapid carbon dioxide production upon thaw. Proc. Natl Acad. Sci. USA.

[14] Vonk JE (2013). High biolability of ancient permafrost carbon upon thaw. Geophys. Res. Lett..

[15] Walter Anthony K (2016). Methane emissions proportional to permafrost carbon thawed in Arctic lakes since the 1950s. Nat. Geosci..

[16] Cooper MDA (2017). Limited contribution of permafrost carbon to methane release from thawing peatlands. Nat. Clim. Change.

[17] Estop-Aragonés C (2018). Limited release of previously-frozen C and increased new peat formation after thaw in permafrost peatlands. Soil Biol. Biochem..

[18] Dyonisius MN (2020). Old carbon reservoirs were not important in the deglacial methane budget. Science.

[19] Elder CD (2019). Seasonal sources of whole‐lake CH_4_ and CO_2_ emissions from interior Alaskan Thermokarst Lakes. J. Geophys. Res. Biogeosciences.

[20] Hicks Pries CE, Schuur EAG, Crummer KG (2013). Thawing permafrost increases old soil and autotrophic respiration in tundra: partitioning ecosystem respiration using δ^13^C and ∆^14^C. Glob. Chang. Biol..

[21] van Huissteden J (2011). Methane emissions from permafrost thaw lakes limited by lake drainage. Nat. Clim. Change.

[22] Weiss N (2016). Thermokarst dynamics and soil organic matter characteristics controlling initial carbon release from permafrost soils in the Siberian Yedoma region. Sediment. Geol..

[23] Muster, S. et al. Size distributions of Arctic waterbodies reveal consistent relations in their statistical moments in space and time. *Front. Earth Sci*. **7**, 5 (2019).

[24] Whiticar MJ (1999). Carbon and hydrogen isotope systematics of bacterial formation and oxidation of methane. Chem. Geol..

[25] Marwick TR (2015). The age of river-transported carbon: a global perspective. Glob. Biogeochem. Cycles.

[26] Drake TW (2018). The ephemeral signature of permafrost carbon in an Arctic fluvial network. J. Geophys. Res. Biogeosci..

[27] Evans CD (2014). Contrasting vulnerability of drained tropical and high-latitude peatlands to fluvial loss of stored carbon. Glob. Biogeochem. Cycles.

[28] Parnell AC (2013). Bayesian stable isotope mixing models. Environmetrics.

[29] Waldron S (2019). C mobilisation in disturbed tropical peat swamps: old DOC can fuel the fluvial efflux of old carbon dioxide, but site recovery can occur. Sci. Rep..

[30] Lambert T, Bouillon S, Darchambeau F, Massicotte P, Borges AV (2016). Shift in the chemical composition of dissolved organic matter in the Congo River network. Biogeosciences.

[31] Cory RM, Kling GW (2018). Interactions between sunlight and microorganisms influence dissolved organic matter degradation along the aquatic continuum. Limnol. Oceanogr. Lett..

[32] Campeau A (2019). Current forest carbon fixation fuels stream CO_2_ emissions. Nat. Commun..

[33] Dean JF (2016). Biogeochemistry of “pristine” freshwater stream and lake systems in the western Canadian Arctic. Biogeochemistry.

[34] Cory RM, Ward CP, Crump BC, Kling GW (2014). Sunlight controls water column processing of carbon in arctic fresh waters. Science.

[35] Stubbins A (2017). Low photolability of yedoma permafrost dissolved organic carbon. J. Geophys. Res. Biogeosci..

[36] Kling GW, Kipphut GW, Miller MC (1991). Arctic lakes and streams as gas conduits to the atmosphere: implications for tundra carbon budgets. Science.

[37] Crawford JT, Striegl RG, Wickland KP, Dornblaser MM, Stanley EH (2013). Emissions of carbon dioxide and methane from a headwater stream network of interior Alaska. J. Geophys. Res. Biogeosci..

[38] Striegl RG, Dornblaser MM, McDonald CP, Rover JR, Stets EG (2012). Carbon dioxide and methane emissions from the Yukon River system. Glob. Biogeochem. Cycles.

[39] Bouchard F (2015). Modern to millennium-old greenhouse gases emitted from ponds and lakes of the Eastern Canadian Arctic (Bylot Island, Nunavut). Biogeosciences.

[40] Wik M, Varner RK, Anthony KW, MacIntyre S, Bastviken D (2016). Climate-sensitive northern lakes and ponds are critical components of methane release. Nat. Geosci..

[41] Bogard MJ (2019). Negligible cycling of terrestrial carbon in many lakes of the arid circumpolar landscape. Nat. Geosci..

[42] Hilton RG (2015). Erosion of organic carbon in the Arctic as a geological carbon dioxide sink. Nature.

[43] Liljedahl AK (2016). Pan-Arctic ice-wedge degradation in warming permafrost and its influence on tundra hydrology. Nat. Geosci..

[44] Parmentier FJW (2011). Spatial and temporal dynamics in eddy covariance observations of methane fluxes at a tundra site in northeastern Siberia. J. Geophys. Res Biogeosciences.

[45] Schaefer H (2016). A 21st-century shift from fossil-fuel to biogenic methane emissions indicated by ^13^CH_4_. Science.

[46] Moran MA, Sheldon WM, Zepp RG (2000). Carbon loss and optical property changes during long-term photochemical and biological degradation of estuarine dissolved organic matter. Limnol. Oceanogr..

[47] Billett MF, Garnett MH, Harvey F (2007). UK peatland streams release old carbon dioxide to the atmosphere and young dissolved organic carbon to rivers. Geophys. Res. Lett..

[48] Walter Anthony KM, Anthony P, Grosse G, Chanton J (2012). Geologic methane seeps along boundaries of Arctic permafrost thaw and melting glaciers. Nat. Geosci..

[49] Budishchev A (2014). Evaluation of a plot scale methane emission model at the ecosystem scale using eddy covariance observations and footprint modelling. Biogeosciences.

[50] Garnett MH, Billett MFF, Gulliver P, Dean JF (2016). A new field approach for the collection of samples for aquatic ^14^CO_2_ analysis using headspace equilibration and molecular sieve traps: the super headspace method. Ecohydrology.

[51] Dean JF, Billett MF, Murray C, Garnett MH (2017). Ancient dissolved methane in inland waters revealed by a new collection method at low field concentrations for radiocarbon (^14^C) analysis. Water Res..

[52] Gulliver P, Waldron S, Scott EM, Bryant CL (2010). The effect of storage on the radiocarbon, stable carbon and nitrogen isotopic signatures and concentrations of riverine DOM. Radiocarbon.

[53] Xu S (2004). Capabilities of the new SUERC 5MV AMS facility for ^14^C dating. Radiocarbon.

[54] Stuiver M, Polach HA (1977). Discussion reporting of ^14^C data. Radiocarbon.

[55] Brass M, Röckmann T (2010). Continuous-flow isotope ratio mass spectrometry method for carbon and hydrogen isotope measurements on atmospheric methane. Atmos. Meas. Tech..

[56] St-Jean G (2003). Automated quantitative and isotopic (^13^C) analysis of dissolved inorganic carbon and dissolved organic carbon in continuous-flow using a total organic carbon analyser. Rapid Commun. Mass Spectrom..

[57] Bouillon S, Korntheuer M, Baeyens W, Dehairs F (2006). A new automated setup for stable isotope analysis of dissolved organic carbon. Limnol. Oceanogr. Methods.

[58] Bröder L, Tesi T, Andersson A, Semiletov I, Gustafsson Ö (2018). Bounding cross-shelf transport time and degradation in Siberian-Arctic land-ocean carbon transfer. Nat. Commun..

[59] Borges AV (2015). Globally significant greenhouse-gas emissions from African inland waters. Nat. Geosci..

[60] Cole JJ, Caraco NF (1998). Atmospheric exchange of carbon dioxide in a low-wind oligotrophic lake measured by the addition of SF 6. Limnol. Oceanogr..

[61] Holgerson MA, Raymond PA (2016). Large contribution to inland water CO_2_ and CH_4_ emissions from very small ponds. Nat. Geosci..

[62] Aufdenkampe AK (2011). Riverine coupling of biogeochemical cycles between land, oceans, and atmosphere. Front. Ecol. Environ..

[63] Aubinet M (1999). Estimates of the annual net carbon and water exchange of forests: the EUROFLUX methodology. Adv. Ecol. Res..

[64] Papale D (2006). Towards a standardized processing of Net Ecosystem Exchange measured with eddy covariance technique: algorithms and uncertainty estimation. Biogeosciences.

