# Publisher Correction: East Siberian Arctic inland waters emit mostly contemporary carbon

**DOI:** 10.1038/s41467-020-16005-1

**Published:** 2020-04-22

**Authors:** Joshua F. Dean, Ove H. Meisel, Melanie Martyn Rosco, Luca Belelli Marchesini, Mark H. Garnett, Henk Lenderink, Richard van Logtestijn, Alberto V. Borges, Steven Bouillon, Thibault Lambert, Thomas Röckmann, Trofim Maximov, Roman Petrov, Sergei Karsanaev, Rien Aerts, Jacobus van Huissteden, Jorien E. Vonk, A. Johannes Dolman

**Affiliations:** 1 Department of Earth Sciences, Vrije Universiteit Amsterdam, Amsterdam, The Netherlands; 2 School of Environmental Sciences, University of Liverpool, Liverpool, UK; 3 Department of Sustainable Agro-Ecosystems and Bioresources, Research and Innovation Centre, Fondazione Edmund Mach, San Michele all’Adige, Italy; 4 Department of Landscape Design and Sustainable Ecosystems, Agrarian-Technological Institute, RUDN University, Moscow, Russia; 5 Natural Environment Research Council Radiocarbon Facility, East Kilbride, UK; 6 Department of Ecological Sciences, Vrije Universiteit Amsterdam, Amsterdam, The Netherlands; 7 Chemical Oceanography Unit, University of Liège, Liège, Belgium; 8 Department of Earth and Environmental Science, Katholieke Universiteit Leuven, Leuven, Belgium; 9 Institute for Marine and Atmospheric Research, Utrecht University, Utrecht, The Netherlands; 10 Institute for Biological Problems of the Cryolithozone, Siberian Branch Russian Academy of Sciences, Yakutsk, Russia; 11 North-Eastern Federal University, Yakutsk, Russia

**Keywords:** Carbon cycle, Cryospheric science

Correction to: *Nature Communications* 10.1038/s41467-020-15511-6, published online 2 April 2020.

The original version of this article contained an error in Fig. 4, in which the *y* axis in the inset ^14^C (pmC) vs mg C L^−1^ graph in Fig. 4c was skewed by ~2 units. This has been corrected in both the PDF and HTML versions of the article.

