# Supplementary material for: East Siberian Arctic inland waters emit mostly contemporary carbon
Source: Nat Commun. 2020 Apr 2;11:1627. doi: 10.1038/s41467-020-15511-6 (PMC7118085; doi:10.1038/s41467-020-15511-6)
Supplement: Supplementary file 1 — Supplementary Information [file 41467_2020_15511_MOESM1_ESM.pdf]

Supplementary information for:

East Siberian Arctic inland waters emit mostly contemporary carbon

Joshua F. Dean *et al*.

Supplementary Methods

General sample collection and carbon concentration analyses. Electrical conductivity, temperature and pH data were collected in situ during sample collection (Greisinger GMH 3431 and 3531 meters); meters were calibrated daily. 100 mL water samples were filtered on the same day as collection with pre-ashed 0.7 µm Whatman GF/F glass fiber filters. Filtrate was kept for DOC analyses, acidified to pH < 2 with HNO_3 and refrigerated prior to analysis (within three months of collection). The GF/F filters were covered and allowed to dry (1-2 days at room temperature) and kept for POC analyses (within three months of collection). Following drying, filters were sealed and checked visually for signs of remaining moisture and dried for longer if needed. Dissolved CO_2 and CH_4 samples were collected using the headspace method whereby 30 mL of sample water was taken from the top 5-10 cm of the water body with a 60 mL syringe and equilibrated with 30 mL of ambient air for 1 min; 12-15 mL of this headspace was then injected into a pre-evacuated 12 mL borosilicate Exetainer® tube with slight over-pressurization (Labco, UK)[33,59]. CO_2/CH_4 concentration samples could not be collected from the Yedoma meltwater due to prohibitive field conditions. Ambient air samples for headspace concentration corrections were collected periodically during sampling and supplemented with data from the eddy covariance tower where needed (Fig. S1).

DOC samples were analyzed by wet oxidation on an Aurora 1030 total carbon analyzer (OI Analytical) in Leuven, Belgium[56,57]. CO_2 generated from DOC oxidation was trapped online by immersion of a stainless-steel loop in liquid N_2 and transferred to a Delta V Advantage IRMS for quantification. Data were calibrated using a series of standards of IAEA-C6 and an in-house sucrose prepared at different concentrations with < ± 5% precision, and checked to ensure completeness of combustion[30]. δ^{13}C-DOC data were consistent across samples collected from the same locations at different time points, and generally consistent overall, indicating negligible microbial activity occurred in the samples between collection and analysis (Data S2). Due to technical issues, only two measurements were made of fluvial samples; however, the between-site relationship seen in Fig. 3A

is consistent with measurements of absorbance at 350 nm (for $n = 6$ fluvial samples; Fig. S4), which is strongly correlated with DOC concentration[46].

GF/F filter samples along with blank filters were subsampled for carbon analysis with stainless steel cylinders (4 to 16 punches with diameter of 3.6mm) and placed in a pre-combusted 5 x 9 mm Ag capsule. Sample capsules were acidified with 1M HCl and dried overnight at 60 °C. Ag capsules were then folded into a tin capsule and analyzed by dry combustion with a Thermo Flash elemental analyzer 1112 series (Thermo Scientific, Rodana, Italy). Aspartic acid was used for calibration and 1-3 replicates were run to assess POC mass on the whole filter; precision was < ± 2%.

CO_2 and CH_4 concentrations were measured using an SRI 8610C Gas Chromatograph with a flame ionization detector. The gas chromatograph was calibrated with CO_2:CH_4:N_2 mixtures (Air Liquide Belgium) of 400, 1000, 4,000 ppm CO_2, and 1, 10 and 30 ppm CH_4. Concentrations were calculated using the standard curves and solubility coefficients, and corrected for ambient atmospheric concentrations in the headspace during collection[59]. Typical precision is ± 2% and ± 3% for CO_2 and CH_4, respectively.

Dissolved organic matter structural characteristics. DOM samples were immediately filtered to 0.2 µm and stored in 20 mL amber glass vials with polytetrafluoroethylene-coated septa[30]. DOM was characterized by absorbance and fluorescence spectroscopy following ref.[30]. Absorbance was measured on a Perkin-Elmer UV/Vis 650S spectrophotometer using a 1 cm quartz cuvette and blank corrected. From this we calculated the ratio of absorption at 250 to 365 nm ($E_2 : E_3$), the ratio of the slope of absorption between 275-295 nm and 350-400 nm (S_R), and absorbance at 350 nm (Data S2). A Perkin-Elmer LS45 fluorescence spectrometer was used to measured fluorescence intensity across excitation and emission wavelengths of 220–450 nm (5 nm increments) and 230–600 nm (0.5 nm increments), respectively. Excitation-emission spectra were analyzed with parallel factor analysis (PARAFAC) to identify abundances of PARAFAC components indicative of DOM structural properties[30] (Data S2).

After excluding DO^{14}C values from the thermokarst lake and Yedoma meltwater following the ^{14}C correlations (Fig. S2), DO^{14}C was significantly correlated with all examined DOM indices, including: S_R (slope ratio) and $E_2 : E_3$ ratio (both inversely proportional to molecular weight), Biological Index (BIX; indicative of biological production contributing to the DOM pool), Humification Index (HIX; indicative of humic content), total fluorescence signal of the DOM pool, percent C1 (proportional contribution of PARAFAC component C1 to the total fluorescence signal, associated with high aromaticity and high molecular weight DOM derived from terrestrial sources), percent C2 (associated with low molecular weight DOM derived from microbial sources), percent C3 (associated with high

aromaticity and high molecular weight DOM derived from terrestrial sources), and percent C5 (associated with low aromaticity and low molecular weight DOM)[30]. The only indices which did not correlate were the Fluorescence Index (indicative of terrestrial versus microbial contributions to the DOM pool) and percent C4 (associated with aromaticity and intermediate molecular weight DOM derived from terrestrial sources; Fig. S6).

Radiocarbon and $\delta^{13}C$. $DO^{14}C$ and $PO^{14}C$ were collected in 500 mL acid-washed HDPE bottles after rinsing with sample water. 500 mL of sample water was filtered on the same day over pre-ashed 0.7 µm Whatman GF/F glass fiber filters and the filtrate kept for $DO^{14}C$ analysis (samples were refrigerated and processed within 2 months of collection), while the filter was covered and allowed to dry (1-2 days) for $PO^{14}C$ analysis. As with the POC concentration samples, the filters were then sealed and checked visually for signs of remaining moisture and dried for longer if needed. CO_2 isotope samples were collected using the super headspace method[50], whereby 3 L of sample water was equilibrated with a 1 L CO_2-free headspace for 3 min, and the headspace injected onto a zeolite molecular sieve cartridge (MSC) – four equilibrations were carried out for each site and injected on a single MSC. CH_4 isotope samples were collected using the coiled membrane vessel (CMV) method[51]. CMVs continuously pumped *in situ* sample water through 6 m of hydrophobic gas-permeable membrane, equilibrating the dissolved gases with an 8 L headspace composed of ambient air; CMVs were left to pump water through the membrane overnight and the headspace split between two 10 L foil gas bags (SKC Ltd, UK) upon collection, and a ~12 mL aliquot collected for $\delta^{13}C$-CH_4 analysis independent from the radiocarbon lab (see below). A $^{14}CH_4$ sample could not be collected from the Yedoma meltwater due to prohibitive field conditions. These collection methods have been shown to minimize contamination during field collection; all samples were processed to CO_2, or extracted from MSCs in the case of the $^{14}CO_2$ samples, within two months of field collection, so storage times and transport should not have impacted the stable or radiogenic isotope signatures of any samples[7,50,52].

CO_2 samples were collected in a headspace scrubbed of ambient CO_2 using a soda lime cartridge[50]. CH_4 samples were collected using an ambient headspace, so were corrected for ambient CH_4 isotopic content using equation S1:

$$Ci_{corr} = \frac{Ci_{meas} - F_{atmos} \cdot Ci_{atmos}}{1 - F_{atmos}} \qquad \text{(S1)}$$

where *Ci* represents the carbon isotope content (^{14}C or ^{13}C) of the corrected sample ($_{corr}$), measured sample ($_{meas}$), and ambient atmospheric values ($_{atmos}$). Values for atmospheric $^{14}CH_4$ (130 ± 5 pmC), $\delta^{13}C$-CH_4 (-47.2 ± 0.2‰), and ambient CH_4 concentrations (2.0 ± 0.2 ppm) were taken from ref.[51]. Headspace CH_4 concentrations were back calculated from the CO_2 volume recovered during sample processing, which was shown to have good agreement with direct measurements of headspace concentration[51]. Aliquots for $\delta^{13}C$-CH_4 stored in the same pre-evacuated 12 mL borosilicate Exetainer® tubes used for CO_2 and CH_4 concentration analyses prior to transport to the UK showed good agreement with the $\delta^{13}C$-CH_4 samples transported in the foil bag samples (Fig. S8).

All ^{14}C samples were processed at the Natural Environment Research Council (NERC) Radiocarbon Facility, East Kilbride, UK. DO^{14}C samples were processed to solids using rotary evaporation, acid fumigated to remove inorganic C, then combusted to CO_2 (ref. [7]). For PO^{14}C, half the filter (one quarter in the case of the Yedoma meltwater sample) was collected and acid fumigated to remove inorganic C, then combusted to CO_2. $^{14}CO_2$ samples were extracted from the MSCs by heating to 425 °C. $^{14}CH_4$ samples in the foil gas bags were first passed through cartridges containing soda lime and type 13X molecular sieve to remove CO_2, then oxidized to CO_2 at 950 °C using a platinum bead catalyst[51]. Once combusted to CO_2 (or desorbed from the MSCs), the produced CO_2 was recovered cryogenically and if enough sample was present (> 1 ml CO_2) then an aliquot was extracted for $\delta^{13}C$ analysis. Known ^{14}C-content standards were processed alongside samples for quality assurance purposes. For PO^{14}C, additional quality assurance tests were carried out on unused pre-combusted filters used for capturing POC material during field filtration: ^{14}C-standard material was added to the unused filters and combusted to CO_2 producing values within a 2σ range of the standards.

The aliquots of recovered CO_2 for $\delta^{13}C$ were analyzed using a dual-inlet isotope ratio mass spectrometer (Thermo Fisher Delta V), with results reported relative to the Vienna Pee Dee Belemnite standard. The IRMS was calibrated using a suite of international standards (NBS19, IAEA C1 and C5, and USGS 24). A single $\delta^{13}C$-DOC value (-22.9 ‰; L01) was inconsistent with the values measured in Leuven from the same site (-26.9 to -27.1 ‰; see DOC methods above) and so was replaced with the median of the three samples analyzed in Leuven from the same location (Data S2). $\delta^{13}C$-CH_4 was also obtained independently from the field aliquots (see above) at the Institute for Marine and Atmospheric Research Utrecht, using continuous-flow IRMS[55]. Three CH_4 samples were too small to obtain $\delta^{13}C$ IRMS aliquots from the foil gas bags, so $\delta^{13}C$-CH_4 values were obtained online via the AMS – these values were only used to normalize the ^{14}C results following convention for which they are suitable, but AMS $\delta^{13}C$ values were not otherwise included in the analyses (Fig. 2).

The CO_2 aliquots for ^{14}C analysis were graphitized using Fe-Zn reduction and the ^{14}C content determined by Accelerator Mass Spectrometry at the Scottish Universities Environmental Research Centre[53]. ^{14}C results were normalized using $\delta^{13}C$ following convention and presented in percent modern carbon (pmC) and conventional ^{14}C ages in years before present (yBP, where 0 yBP. = 1950 CE) [54]. Known age standards were processed alongside ^{14}C samples using identical processing methods for quality assurance.

Supplementary Figures

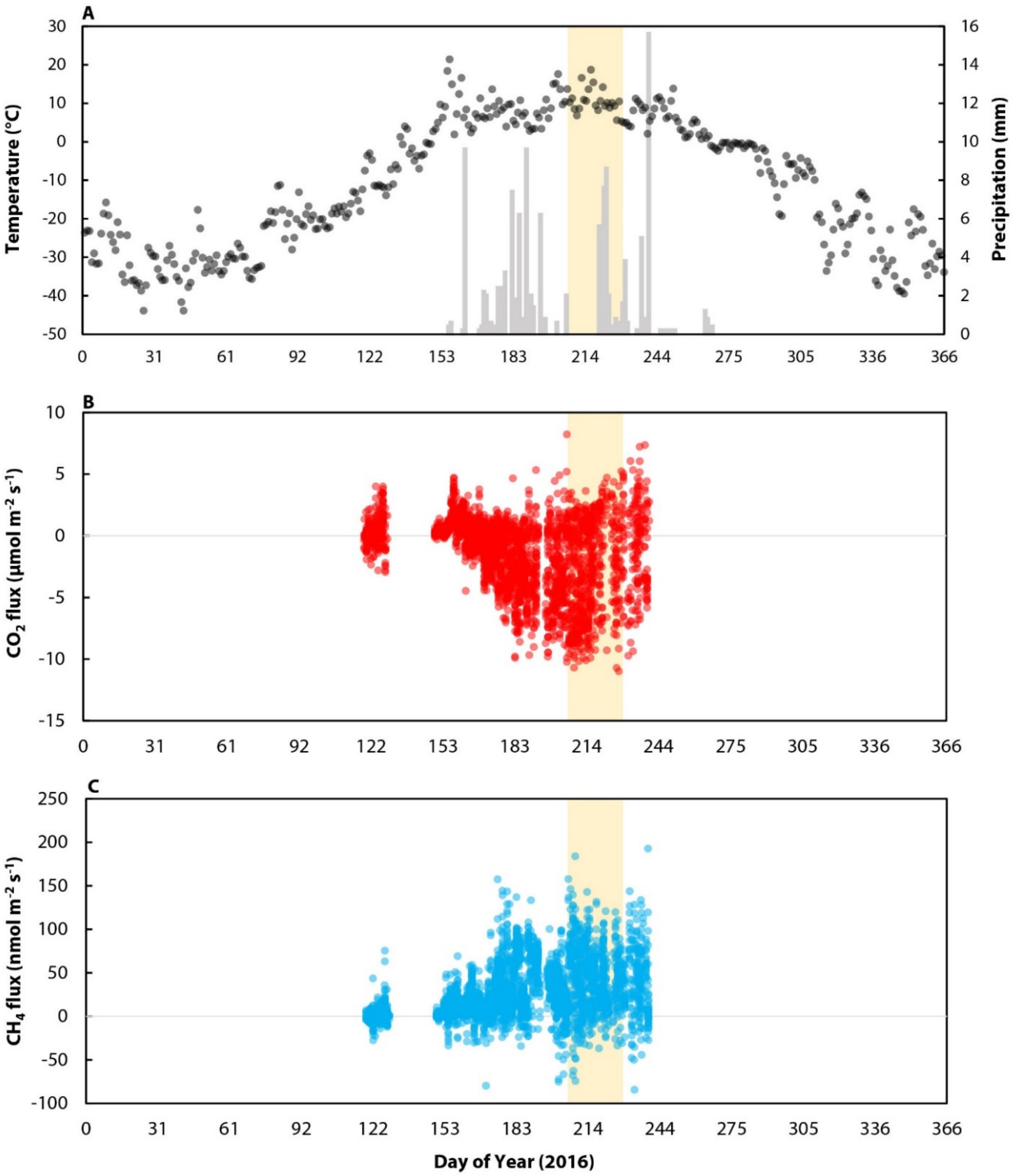

Supplementary Figure 1. Precipitation and air temperature variability, and carbon dioxide (CO_2) and methane (CH_4) fluxes for the study year: 2016. (A) Daily temperature (black dots) and precipitation (grey bars), and available 30-min tundra **(B)** CO_2 and **(C)** CH_4 flux data from the eddy covariance tower at the study site for 2016. The study period is indicated by the shaded area (25 July 2016 to 17 August 2016; Day of Year 206-229).

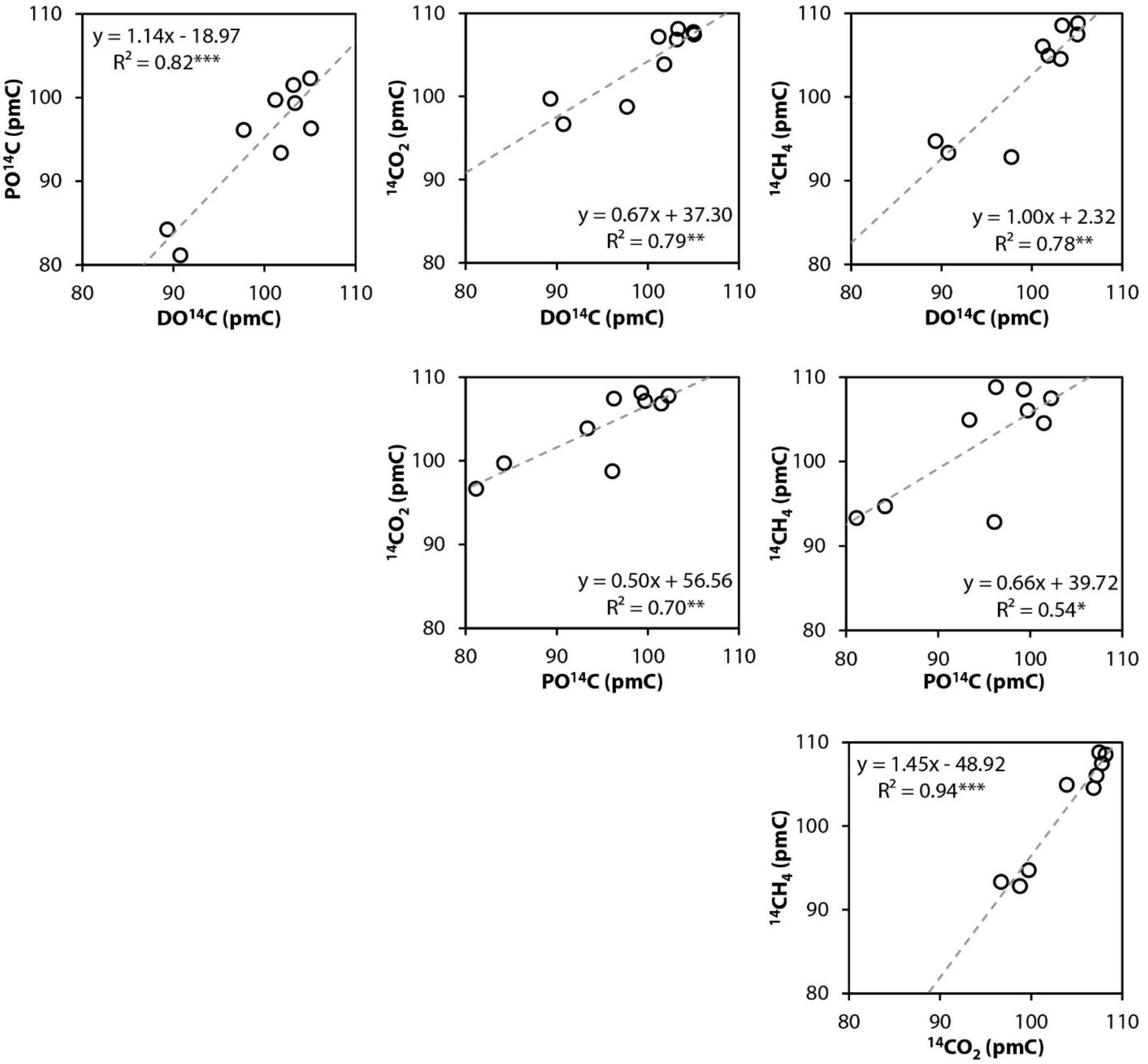

Supplementary Figure 2. Correlations between radiocarbon (^{14}C) signatures in the different inland water carbon components excluding thermokarst lake and Yedoma samples. *P*-values are indicated by asterisks alongside the R^2-values ($*p < 0.05$, $**p < 0.01$, $***p < 0.001$).

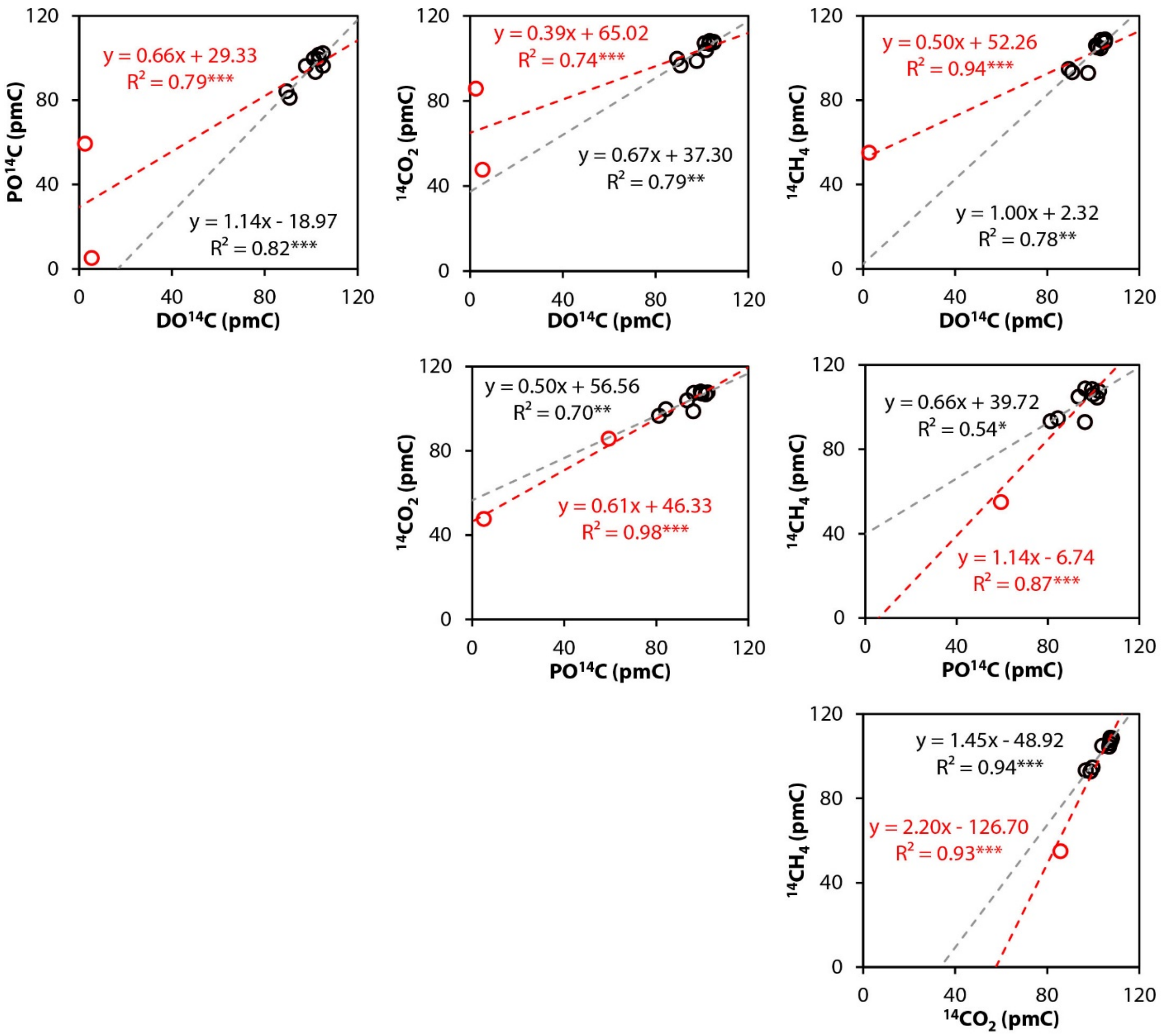

Supplementary Figure 3. Full correlations between radiocarbon (^{14}C) signatures in the different inland water carbon components. Excluding the thermokarst lake and Yedoma samples as in Fig. S2 (black points, line equations and R^2-values), and with all data (red points, line equations and R^2-values). *P*-values are indicated by asterisks alongside the R^2-values (*$p < 0.05$, **$p < 0.01$, ***$p < 0.001$).

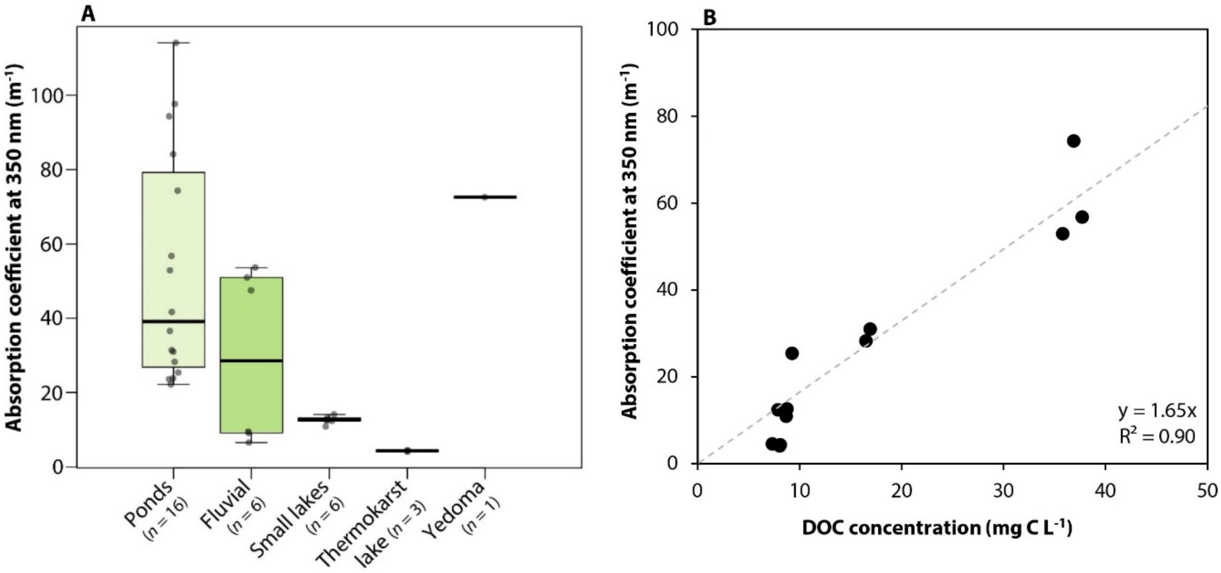

Supplementary Figure 4. Absorbance coefficient at 350 nm (a350) as a proxy for dissolved organic carbon (DOC) concentration. (A) a350 values for each sampling site; the thick horizontal lines represent the median, limits of the boxes represent upper and lower quartiles, whiskers extend to 1.5 times the interquartile range, dots represent all data points. **(B)** Relationship between a350 and DOC concentration for all available samples.

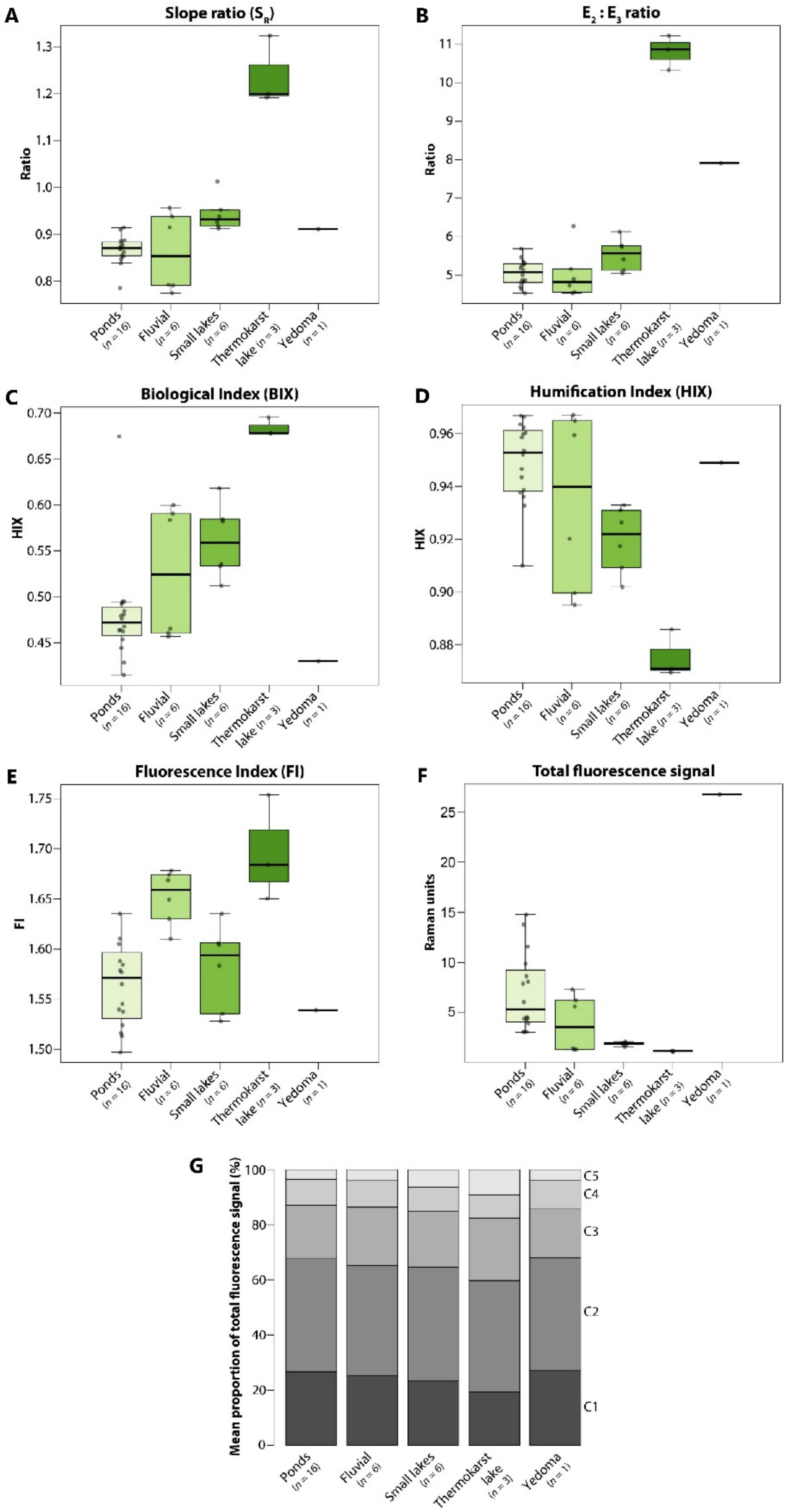

Supplementary Figure 5. Dissolved Organic Matter (DOM) structural indices for each sampling location. **(A)** Slope ratio (S_R); **(B)** $E_2 : E_3$ ratio; **(C)** Biological Index (BIX); **(D)** Humification Index (HIX); **(E)** Fluorescence Index (FI); **(F)** the total fluorescence signal; thick horizontal lines represent the median, limits of boxes represent upper and lower quartiles, whiskers extend to 1.5 times the interquartile range, dots represent all data points. **(G)** proportional contribution of each modelled parallel factor analysis (PARAFAC) component (C*x*) to the total fluorescence signal.

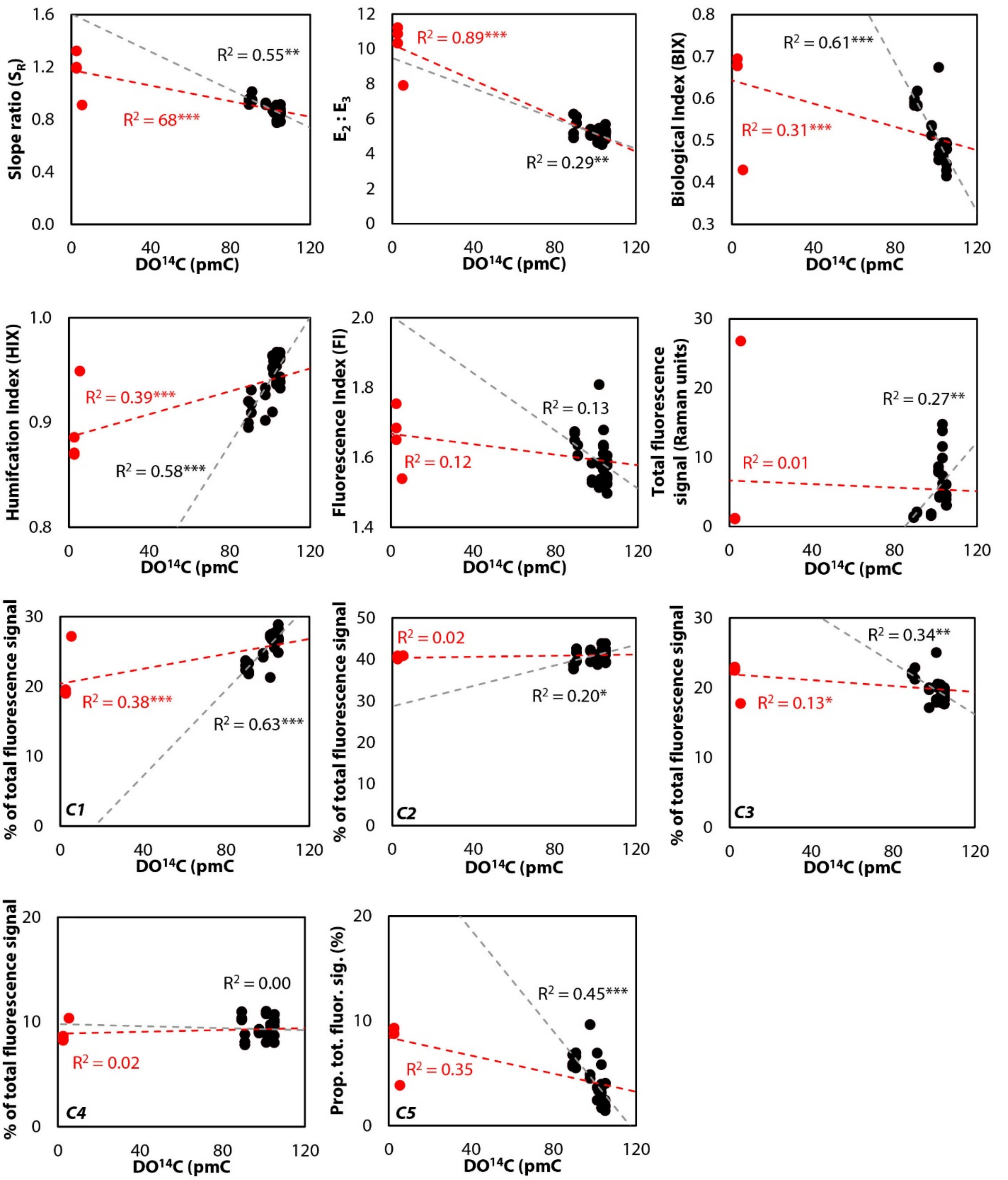

Supplementary Figure 6. Correlations between the radiocarbon content of dissolved organic carbon ($DO^{14}C$) and the dissolved organic matter (DOM) structural indices. The black line and black R^2-values correspond to the correlations excluding the thermokarst lake and Yedoma $DO^{14}C$ values; the red line and red R^2-values correspond to the correlations including all data. *P*-values are indicated by asterisks alongside the R^2-values (**p* < 0.05, ***p* < 0.01, ****p* < 0.001).

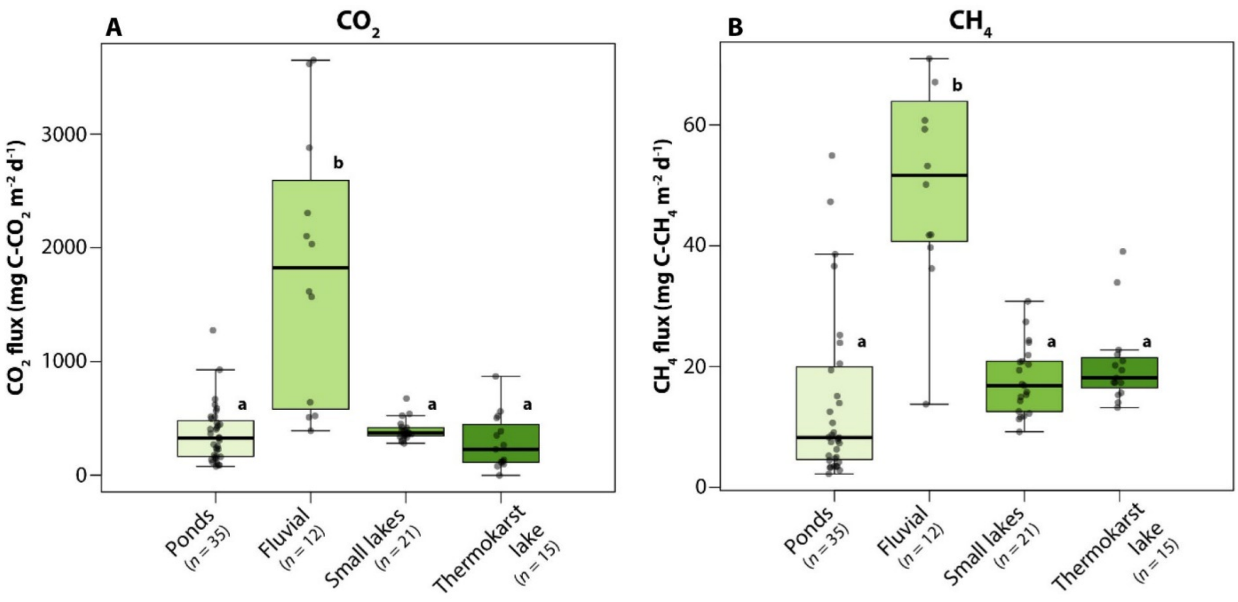

Supplementary Figure 7. (A) Carbon dioxide (CO_2) and (B) methane (CH_4) emissions from the inland water sites. The thick horizontal lines represent the median, limits of the boxes represent upper and lower quartiles, whiskers extend to 1.5 times the interquartile range, dots represent all data points; letters indicate statistical differences between the sample locations using ANOVA (see Methods).

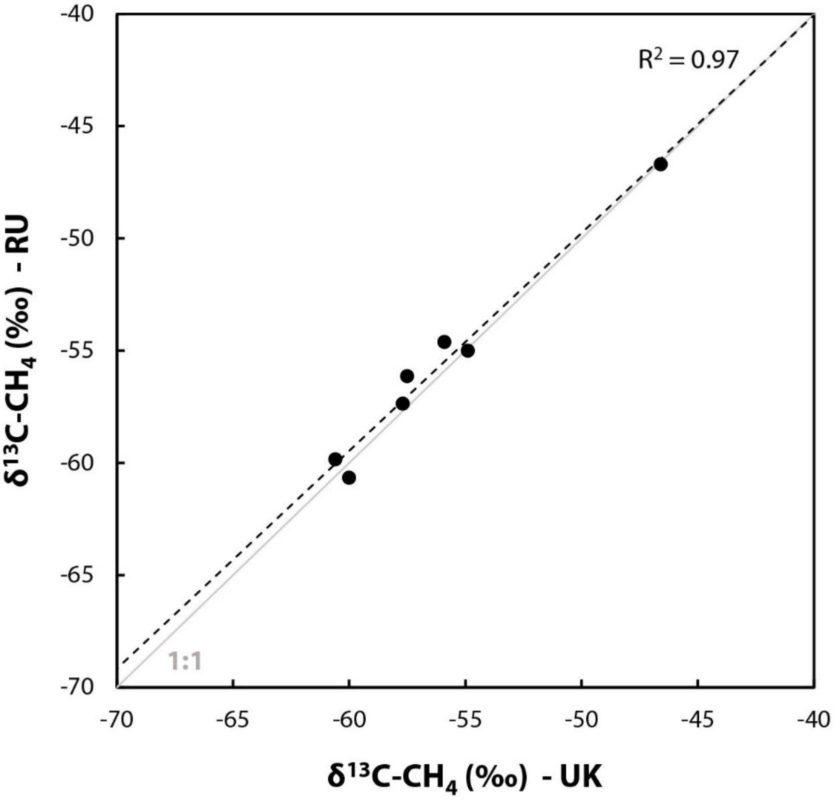

Supplementary Figure 8. Comparison of δ^{13}C-CH_4 isotopes collected before and after transport to the UK Radiocarbon Facility. Values on the *x*-axis represent samples transported to the UK in foil gas bags, values on the *y*-axis represent aliquots injected into in pre-evacuated 12 mL borosilicate Exetainer® tubes in the field and analyzed at the Institute for Marine and Atmospheric Research Utrecht[55]. The dashed black line (R^2-value in black text) shows good agreement between the values compared with a 1:1 relationship (solid grey line).

Supplementary Tables

Supplementary Table 1. Field and descriptive parameters for each site (Fig. 1).

Site ID	Sample location	Temperature (°C) [range; *n* = 3]	EC (μS cm^{-1}) [range; *n* = 3]	pH [range; *n* = 3]	Surface area (m^2)	Active layer depth (m)	Water depth (m) [± 1σ]
P01*	ponds	10.8-21.8	37-41	5.6-5.8	96	0.38	0.40
P02*	ponds	11.3-22.4	15-16	5.9-6.1	36	0.48	0.20
P03*	ponds	11.3-21.9	15-18	6.1-6.3	156	0.51	0.20
P04	ponds	11.3-21.7	15-17	6.0-6.7	165	0.39	0.20
P05	ponds	10.4-21.0	20-26	5.5-5.9	96	0.36	0.22
P06	ponds	9.1-17.4	25-28	5.1-5.3	3	0.48	0.12
P07	ponds	10.6-17.8	15-16	5.7-6.2	56	0.52	0.20
P08*	ponds	10.8-15.1	24-33	5.9-6.6	144	0.50	0.30
P09	ponds	11.0-14.3	23-32	5.9-6.3	240	0.43	0.40
P10	ponds	10.1-17.5	42-52	5.4-5.6	32	0.45	0.15
P11*	ponds	10.3-15.3	23-25	5.3-5.5	60	0.46	0.30
P12	ponds	10.5-13.7	23-24	5.3-5.4	117	-	0.20
S01*	fluvial	13.5-14.7	46-49	6.2-7.1	60	-	0.30
S02	fluvial	14.8-14.9	47-50	6.9-7.8	-	-	-
S03*	fluvial	13.7-15.4	50-77	7.2-7.6	-	-	-
S04	fluvial	14.2-15.4	46-54	7.2-7.5	-	-	-
L06	small lakes	12.2-13.7	29-34	6.9-7.3	140597	-	1.61 ± 0.35
L07	small lakes	12.0-13.6	28-29	6.7-7.4	140597	-	1.61 ± 0.35
L08*	small lakes	12.3-13.7	28-30	6.6-7.3	140597	-	1.61 ± 0.35
L09	small lakes	12.9-13.8	28-30	6.6-7.3	140597	-	1.61 ± 0.35
L10*	small lakes	13.0-13.8	16-17	6.3-6.9	41719	-	2.04 ± 0.65
L11	small lakes	13.2-13.8	16-17	6.0-6.9	41719	-	2.04 ± 0.65
L12	small lakes	13.1-13.8	16-17	6.3-7.0	41719	-	2.04 ± 0.65
L01*	thermokarst lake	14.4-14.8	203-205	7.6-7.9	511189	-	3.06 ± 1.18
L02	thermokarst lake	14.8-15.9	200-204	8.1-8.2	511189	-	3.06 ± 1.18
L03	thermokarst lake	14.2-17.4	204-209	7.8-8.0	511189	-	3.06 ± 1.18
L04	thermokarst lake	14.4-15.6	200-203	7.9-8.2	511189	-	3.06 ± 1.18
L05	thermokarst lake	14.3-14.7	201-203	8.1-8.4	511189	-	3.06 ± 1.18

* Sites where radiocarbon (^{14}C) samples were collected.

Supplementary Table 2. Five source isotope mass balance model output. Relative contributions of the five sources used in the isotope mixing model to the radiocarbon (^{14}C) samples collected from each sampling location using a Bayesian statistical source apportionment model (Fig. 3; see Methods).

**Source**	**Ponds (mean ± σ)**	**Fluvial (mean ± σ)**	**Small lakes (mean ± σ)**	**Thermokarst lake (mean ± σ)**	**Yedoma meltwater (mean ± σ)**
		Total carbon (DOC, POC, CO_2 and CH_4)			
Modern	0.73 ± 0.10	0.32 ± 0.22	0.37 ± 0.21	0.17 ± 0.13	0.16 ± 0.14
Topsoil	0.16 ± 0.06	0.31 ± 0.16	0.19 ± 0.12	0.15 ± 0.12	0.15 ± 0.13
Basal peat	0.06 ± 0.06	0.21 ± 0.17	0.27 ± 0.21	0.18 ± 0.15	0.16 ± 0.14
Old	0.03 ± 0.02	0.09 ± 0.07	0.10 ± 0.07	0.24 ± 0.18	0.20 ± 0.17
Ancient	0.02 ± 0.01	0.06 ± 0.04	0.07 ± 0.04	0.26 ± 0.17	0.33 ± 0.23
		Carbon emissions (CO_2 and CH_4)			
Modern	0.68 ± 0.16	0.30 ± 0.22	0.44 ± 0.24	0.19 ± 0.15	0.11 ± 0.08
Topsoil	0.21 ± 0.10	0.33 ± 0.19	0.18 ± 0.14	0.19 ± 0.14	0.11 ± 0.07
Basal peat	0.07 ± 0.07	0.20 ± 0.16	0.22 ± 0.18	0.20 ± 0.16	0.13 ± 0.09
Old	0.03 ± 0.02	0.10 ± 0.09	0.10 ± 0.07	0.22 ± 0.17	0.24 ± 0.20
Ancient	0.02 ± 0.02	0.07 ± 0.06	0.06 ± 0.04	0.20 ± 0.14	0.41 ± 0.14
