# Supplementary material for: East Siberian Arctic inland waters emit mostly contemporary carbon
Source: Nat Commun. 2020 Apr 2;11:1627. doi: 10.1038/s41467-020-15511-6 (PMC7118085; doi:10.1038/s41467-020-15511-6)
Supplement: Supplementary file 3 — Description of Additional Supplementary Files [file 41467_2020_15511_MOESM3_ESM.docx]

**Description of Additional Supplementary Files**

File name: Supplementary Data 1
Description: Isotope values and radiocarbon reporting. Full radiocarbon sample collection details, including publication codes, CO2 volumes recovered from each sample, δ13C and 14C content, radiocarbon ages, and associated uncertainties; note the uncertainties reflect the laboratory analytical uncertainty (known 14C-content standards were processed alongside samples for quality assurance and to verify analytical uncertainties). For the CH4 isotopes additional uncertainties associated with the ambient headspace correction are also incorporated (see Methods).

File name: Supplementary Data 2
Description: Full carbon concentration, dissolved organic matter indices, and carbon dioxide (CO2) and methane (CH4) emission data. Carbon concentration, CO2 and CH4 emission and dissolved organic matter (DOM) data for all samples collected in this study (including δ13C-DOC values from Leuven, see Methods).
