# Supplementary material for: East Siberian Arctic inland waters emit mostly contemporary carbon
Source: Nat Commun. 2020 Apr 2;11:1627. doi: 10.1038/s41467-020-15511-6 (PMC7118085; doi:10.1038/s41467-020-15511-6)
Supplement: Supplementary file 2 — Peer Review File [file 41467_2020_15511_MOESM2_ESM.pdf]

Reviewers' comments:

Reviewer #1 (Remarks to the Author):

Summary

Dean et al. provide a thorough evaluation of the sources and fluxes of CO_2 and CH_4 from a landscape underlain by continuous permafrost in Siberia. Their objective is to determine to what extent modern and “pre-aged” (Holocene and Pleistocene) carbon contribute to C fluxes in surface waters either isolated from or receiving inputs from pre-aged sources. Their approach is to use C concentrations, C isotopes (13C and 14C), and spectroscopic methods to date/characterize different C components in inland waters. One particular strength of this study is that the authors use flux calculations (CO_2 and CH_4 emissions from each water body multiplied by areal extent) to scale their observations to the landscape. In this way, they calculated how much of the C emitted from inland water was derived from contemporary versus pre-aged sources. They found that most of the emitted C derived from contemporary sources, and degradation of pre-aged sources was only substantial (>50%) where waters were impacted by abrupt thaw (thermokarst/Yedoma meltwater).

I am supportive of publication provided the authors address the revisions requested below. The methods are thorough, careful, and well-described. The data analysis is robust and the results are supported by the data. The approach provides important insight into how C is emitted through inland water bodies, which is of interest to a broad audience. Although not revolutionary in thought, the study provides a strong dataset with a fair attempt made to relate the scalability of the results to broader environments.

Major comments

Although it is important to consider the source of C emissions, these inland water emissions are a small fraction of the total C uptake for the system, and even small relative to methane emissions from the tundra. It’s important to emphasize why these inland waters are important to consider relative to the tundra, and how processes in the tundra may influence the signals observed in the inland waters. Is there any evidence for the age of the C emitted from the tundra for comparison to the waters, if not from this study, then compiled from other studies?

It looks like the contemporary C could be derived solely from the active layer, but the mixing model includes non-negligible old and ancient components. Are these reasonable values? Where would the pre-aged C come from if these are isolated from those sources? I am not well versed in the methods used in this single isotope mixing model, so another reviewer more knowledgeable of this method will need to comment.

Specific comments:

l. 36. This line is a bit unclear. Suggest “We show that >80% of total inland water carbon emissions were contemporary in age, but that pre-aged carbon contributed >50% at sites …” in order to differentiate between C content and C emissions. It is possible that this does refer to carbon concentrations, but that was harder to determine from the reported data. I used the data in Table 1 to confirm that >80% of emissions were contemporary, assuming that the contemporary and pre-aged proportions in the last two columns referred to emissions and not the concentrations. It is important to clearly differentiate what these proportions refer to since concentration (DOC + POC + CO_2 + CH_4) is different than emissions (CO_2 + CH_4 fluxes).

l. 41. The use of Mg C for net carbon sink but % for emissions is confusing, particularly the “offset” wording. Suggest “The study region was a net carbon sink (-876.9 …), but inland waters were a source of contemporary (16.8 Mg C) and pre-aged (3.7 Mg C) emissions that respectively offset 1.9 ±

1.2% and 0.4 ± 0.3% of CO_2 uptake by tundra (-897 ± 115 Mg C).

l. 107. “High 13δC-CO_2 values are likely due to fractionation during methanogenesis.” Methane is only a small fraction of the emitted C. Shouldn’t the 13δC-CO_2 values reflect respiration?

l. 120. The statement “DOC values…were consistent with organic matter in general” is super vague. Does that indicate soil organic matter? Living organic matter? Certain types of vegetation? People?

l. 132. Those calculations still leave ~17% pre-aged C. Is there permafrost thaw in the region that could account for that? Is this an artifact of the isotope mixing model? How does the thaw depth correspond to depths for the soil sources presented in Figure 1?

l. 208. Yes, I think this paragraph makes an important point. Incubations are essentially cut off from new C inputs and destabilize soils through homogenization, thereby increasing the potential for older C to be respired. Source is also important – if a stream is draining an older source, it will have an older signal. That is perhaps why the scaling in this paper is so important to the overall interpretation of inland C emissions.

l. 247. It is also necessary to emphasize here that erosional processes are potentially more important during other times of the year, or during abrupt thaw events. This study occurred only for a short and stable period of the summer, and the proportions of the different C sources cannot be assumed constant throughout the year.

Figure 1. Adding the ranges for δ13C sources/pathways described in lines 107-121 to the x-axis, similar to how 14C sources are provided on the y-axes, would be informative for interpreting the graph.

Figure 2. How many data points were included here? Can you include n values for each bar? It looks like there are far more data points than indicated in Figure 3.

Table 1. I’m not familiar with the superscript (+) and subscript (-) in the proportion derived from contemporary sources column. Does that refer to positive and negative uncertainty being different in magnitude?

Supporting info:
The data tables were not provided in the review material and could not be assessed.

Reviewer #2 (Remarks to the Author):

Review of: Arctic inland waters emit mostly contemporary carbon

Synopsis:

The authors present a unique and in-depth geochemical analysis (focusing on carbon isotopes) of the four primary carbon pools that effect greenhouse gas emissions from inland waters in a 16km2 study region in north eastern Siberia. The authors show that on the scale of the whole study region, contemporary (i.e. young) carbon sources play a predominant role over ancient permafrost carbon sources in fueling POC, DOC, CH_4, CO_2 emissions from a variety of inland water types. Using an eddy covariance approach, the authors show that despite high concentrations of dissolved carbon species in the aquatic system, the whole study region was a strong sink of atmospheric CO_2. Given this, and the isotopic evidence, the authors conclude that inland waters in permafrost regions (including those with active thermokarst) are mostly regulated by contemporary carbon turnover compared to isolated

locations of ancient permafrost carbon release. The study is unique in the sense that it targets dissolved carbon pools which, especially for CH4, are more spatially and temporally integrated than ebullition sources. Furthermore, it provides critical insight into an understudied environment that is undergoing rapid change in response to Arctic amplification. Given the uniqueness of the data, I recommend acceptance of the manuscript, but not before several critical issues are addressed. Please find my general comments and line-by-line comments below.

General Comments:

• Title is too broad given the limited scope of the study region. In its current form, the title describes the whole Arctic, yet there are individual Arctic lakes that are larger than the defined study area. I would suggest something slightly more representative of the scope of the study. For example: "Inland waters of a Siberian watershed emit mostly contemporary carbon"
• The introduction has too few details about the study site, methods, and approach. More detail is found later on in the methods section; however, I think it would be helpful to for the reader to have more context about the study before jumping in to the results. It would also be helpful if Figure S1 was brought in the main text as Figure 1. Can the probable footprint of the EC tower be added to the figure?
• The methods for upscaling EC-tower-based fluxes and assigning relative source proportions to isotopically-defined carbon pools seems somewhat arbitrary. A short straightforward description at lease belongs in the main methods section.
• The authors should emphasize that dissolved CH4 pools are more spatiotemporally integrated, and thus more representative, than ebullitive CH4.
• Some paragraphs required re-reading due to long sentences. Some edits below include suggestions to improve clarity.

Specific Comments:

50 – 52: This the certainty of this statement seems to refute lines 33-34 in the abstract that pose this question as a major unknown. No specific recommendation here, but it left me wondering how unknown this topic really is.

61: Oxford comma after "…including CO2)" since CH4 is organic?

78 – 79: Make "a selection of inland water sites" more descriptive and quantitative. See general comment about lack of detail in the introduction.

82: What does relatively undisturbed mean? A more detailed description of the site would be helpful. See general comment about detail in the introduction.

86 – 88: I would speculate that very little is known about the seasonality of C emission sources, especially in thermokarst lakes with substantial taliks. One could argue that the oldest emissions of CH4 in thermokarst lakes occur in the fall or even into the winter as the seasonal heat pulse lags through the deeper thawed sediments. See (Elder et al. 2019 JGR: Biogeosciences) which sites evidence of pre-aged CH4 additions to the below-ice dissolved pool occurring throughout the winter season. The authors state that they likely observed the oldest emissions during just the growing season, however this isn't supported by data and is speculation.

107: Specify the methanogenic pathway that is implied here, since that matters for the δ13C value found in coexisting CO2. Also, can an actual value replace "high values" here to be more quantitative and informative?

107 – 109: The use of "high values" and "less negative" in these lines is a bit confusing. Does "high" reflect "highly negative?" or high as in very heavy δ13C values? I would suggest choosing a

convention and sticking to it (i.e. "very light/very heavy").

109 – 113: Carbonate sediments/minerals can contribute relatively enriched (heavy CO_2) to inland waters. (And 14C-depleted CO_2 for that matter) Is there any evidence of carbonate minerals at this site? Should be specified.

113 – 115: Residual dissolved CH_4 pools are also susceptible to enrichment due to partial oxidation/consumption. If thermogenic sources can be ruled out, this is another potential explanation for the relatively heavy $\delta^{13}CH_4$ values.

154: Define PARAFAC

193 – 195: This seems like a blanket statement that doesn't hold much meaning. Or is at least a little confusing. Do the authors mean to say that given the predominance of contemporary C in inland waters, these systems are influenced more by the contemporary carbon cycle than the cycling of pre-aged C release? Perhaps some wordsmithing would make this a clearer conclusion.

200: Harsh transition at the beginning of this sentence makes the paragraph hard to follow. Perhaps include "However, the high proportion…"

207: Or that the most labile DOC pools are also the youngest. I think "mobilized carbon" in this sentence applies to all POC/DOC.

213: At a faster rate or in greater proportion. Not sure if authors can differentiate rates vs. magnitudes of decomposition of the different OC source pools.

213 – 216: Right. Perhaps wrap this idea back around by concluding this paragraph with a statement that young DOC is either decomposed faster or in greater proportion than pre-aged DOC.

235: Reference 37 makes no mention of the 14C age of ebullition CH_4. Few studies have compared the 14C age of CH_4 in the dissolved pool and ebullition simultaneously (from the same system), However some evidence suggests that $^{14}CH_4$ ebullition on whole-lake scales can resemble that of the dissolved pool within the same lake, and that a large portion of the dissolved CH_4 pool likely originates from ebullition that is trapped and re-dissolved during the ice-cover period. See Elder et al. 2019 Biogeosciences.

237: Add semicolon or even a period after "depth."

239 – 240: It seems that seasonal changes in 14C of dissolved CO_2 is a very different process than what is being described in this section. Not sure how that is a "same pattern" result, especially since this study did not make seasonal measurements. Either this sentence is out of place, or more description is needed to make that connection.

242: It is also possible that older, trapped ebullition-CH_4 is partially oxidized to CO_2 below ice- an "aging" effect on the winter dissolved CO_2 pool. Elder et al. 2019

243 – 246: This is hard to follow- possibly split up into two sentences. See comments above regarding the seasonality of 14C signatures of C emissions. This section is speculation that is not supported by data or reference to other research.

258 – 259: See comment about de-scoping the title of the paper to more appropriately describe the scope of the study region.

265 – 266: But contemporary carbon seems to be the predominant player.

347 – 350: Where were lake and pond samples taken? Edges of the water features or from the shoreline? Depending on the heterogeneity of the lake/pond environment, this can have implications for carbon source variability. This should be discussed.

Supplementary Methods Comments:

46 – 47: Was there a method to check for completeness of combustion? Whether there was or not, this should probably be discussed since there can be significant effects on 14C & 13C results.

160: What is the precision of the 13C measurements from the AMS?

179: Is “subsidized” the right word here?

222: The selection of the upscaled flux region seems arbitrarily chosen to encompass discrete sampling sites. Should it be determined with footprint analysis instead? What is the footprint of the EC tower? This could have drastic implications for the relative proportions of carbon sources assigned to different flux areas.

References:

Elder, C. D., M. Schweiger, B. Lam, E. D. Crook, X. Xu, J. Walker, K. M. Walter Anthony, and C. I. Czimczik. "Seasonal Sources of Whole-Lake CH_4 and CO_2 Emissions From Interior Alaskan Thermokarst Lakes." Journal of Geophysical Research: Biogeosciences (2019).

Reviewer #1 (Remarks to the Author):

Summary

Dean et al. provide a thorough evaluation of the sources and fluxes of CO2 and CH4 from a landscape underlain by continuous permafrost in Siberia. Their objective is to determine to what extent modern and "pre-aged" (Holocene and Pleistocene) carbon contribute to C fluxes in surface waters either isolated from or receiving inputs from pre-aged sources. Their approach is to use C concentrations, C isotopes (13C and 14C), and spectroscopic methods to date/characterize different C components in inland waters. One particular strength of this study is that the authors use flux calculations (CO2 and CH4 emissions from each water body multiplied by areal extent) to scale their observations to the landscape. In this way, they calculated how much of the C emitted from inland water was derived from contemporary versus pre-aged sources. They found that most of the emitted C derived from contemporary sources, and degradation of pre-aged sources was only substantial (>50%) where waters were impacted by abrupt thaw (thermokarst/Yedoma meltwater).

I am supportive of publication provided the authors address the revisions requested below. The methods are thorough, careful, and well-described. The data analysis is robust and the results are supported by the data. The approach provides important insight into how C is emitted through inland water bodies, which is of interest to a broad audience. Although not revolutionary in thought, the study provides a strong dataset with a fair attempt made to relate the scalability of the results to broader environments.

We thank the reviewer for their positive comments, and we appreciate their perspective on the challenges of scalability and that we were able to satisfactorily address this in our study. These comments were helpful in producing what we feel is a stronger manuscript.

Major comments

Although it is important to consider the source of C emissions, these inland water emissions are a small fraction of the total C uptake for the system, and even small relative to methane emissions from the tundra. It's important to emphasize why these inland waters are important to consider relative to the tundra, and how processes in the tundra may influence the signals observed in the inland waters. Is there any evidence for the age of the C emitted from the tundra for comparison to the waters, if not from this study, then compiled from other studies?

We emphasise in the introduction that inland waters are recognised as important areas of carbon emissions in many landscapes (L.47). Recent high-impact papers have suggested that rivers and lakes in the Arctic can be large emitters of carbon (e.g. Serikova et al., 2018 *Nature Geoscience* doi: 10.1038/s41561-018-0218-1) but these studies rarely provide context by measuring terrestrial fluxes at the same time. This is largely an issue of scale, but we have attempted to provide context here for our measurements. Unfortunately, there is no ^{14}C data for soil emission from the Siberian Arctic. We only found one study which used lab incubations to indirectly show that thawing Siberian Yedoma soils can release old CO_2 (Dutta et al., 2006 Global *Change Biology* doi: 10.1111/j.1365-2486.2006.01259.x). The isotopic signature of soil CO_2 and CH_4 from soils in this large region of the Arctic is therefore a major knowledge gap.

It looks like the contemporary C could be derived solely from the active layer, but the mixing model includes non-negligible old and ancient components. Are these reasonable values? Where would the pre-aged C come from if these are isolated from those sources? I am not well versed in the methods

used in this single isotope mixing model, so another reviewer more knowledgeable of this method will need to comment.

The mixing model is a statistical representation of all the potential contributions of different soil components. For consistency, we used the same end-members for all the analyses, rather than trying to justify different end-members for individual water types. The different solutions include the potential for carbon to be derived exclusively from the active layer. The medians shown in Fig. 3 and Table 2 represent the middle ground of all these solutions, but are ultimately indicative rather than absolute. We felt it more important to represent all the possible contributions that could be occurring, even though it is unlikely that old and ancient carbon is available in the ponds, for example (see L.83-94). Either way, the difference in ages and possible source contributions are quite clear in Fig. 3 and look similar to previous work (e.g. Elder et al., 2018 *Nature Climate Change* doi: 10.1038/s41558-017-0066-9; Dean et al., 2018 *Environmental Research Letters* doi: 10.1088/1748-9326/aaa1fe). We now address this specifically in the introduction (L.83-94).

Specific comments:

l. 36. This line is a bit unclear. Suggest "We show that >80% of total inland water carbon emissions were contemporary in age, but that pre-aged carbon contributed >50% at sites …" in order to differentiate between C content and C emissions. It is possible that this does refer to carbon concentrations, but that was harder to determine from the reported data. I used the data in Table 1 to confirm that >80% of emissions were contemporary, assuming that the contemporary and pre-aged proportions in the last two columns referred to emissions and not the concentrations. It is important to clearly differentiate what these proportions refer to since concentration (DOC + POC + CO2 + CH4) is different than emissions (CO2 + CH4 fluxes).

Please see response to L.41 below.

l. 41. The use of Mg C for net carbon sink but % for emissions is confusing, particularly the "offset" wording. Suggest "The study region was a net carbon sink (-876.9 …), but inland waters were a source of contemporary (16.8 Mg C) and pre-aged (3.7 Mg C) emissions that respectively offset 1.9 ± 1.2% and 0.4 ± 0.3% of CO2 uptake by tundra (-897 ± 115 Mg C).

We thank the reviewer for highlighting these calculations. Upon revisiting them, we realise that the description of these calculations was a little confusing and the reviewer comments prompted us to clarify this in Table 1, Table S2 and in the text. Table 1 and Fig. 3 show the proportional contributions of contemporary and pre-aged sources to all carbon forms, and Table S2 shows proportional contributions of all five sources for all carbon forms <u>and</u> just for CO_2 and CH_4. The latter (CO_2 and CH_4 source contributions) are now used to calculate emissions of contemporary versus pre-aged carbon as reported in the updated text (L.200-204). We updated L.36 to highlight that those proportions refer to the total carbon concentrations – please note the relative source contributions for total carbon versus just the CO_2 and CH_4 were very similar (Table S2): contemporary fluxes in previous manuscript = 16.8 ± 10.4 versus 17.0 ± 10.9 Mg C in the updated manuscript; pre-aged fluxes in the previous manuscript = 3.6 ± 2.8 versus 3.5 ± 2.3 Mg C in the updated manuscript. Please note we ultimately deleted the sentence this comment refers to in order to meet the length requirements for the abstract in *Nature Communications*, but the calculations are included in the text as indicated above.

l. 107. "High 13δC-CO2 values are likely due to fractionation during methanogenesis." Methane is only a small fraction of the emitted C. Shouldn't the 13δC-CO2 values reflect respiration?

We have updated the text here to highlight that the $\delta^{13}C\text{-}CO_2$ values represent a mixture of respiration and potentially methanogenesis as well (L.122-123).

l. 120. The statement "DOC values…were consistent with organic matter in general" is super vague. Does that indicate soil organic matter? Living organic matter? Certain types of vegetation? People?

We have reworded this to be more specific: "… were consistent with freshwater DOC derived from the C3 photosynthetic pathway" (L.138-139).

l. 132. Those calculations still leave ~17% pre-aged C. Is there permafrost thaw in the region that could account for that? Is this an artifact of the isotope mixing model? How does the thaw depth correspond to depths for the soil sources presented in Figure 1?

Please see our response to this reviewer's earlier comment on this. This is largely an artefact of the mixing model, with it being unlikely that there are substantial contributions of pre-aged carbon to some waters, especially the ponds. We chose to include all sources in the model for consistency and to explore the potential contributions that could produce the observed ^{14}C values. This is now specifically mentioned in the introduction (L.83-94). The key point is that the observed inland water carbon is primarily from contemporary sources, except for the thermokarst lake and Yedoma samples, which is clear in Table 1 and Fig. 3.

l. 208. Yes, I think this paragraph makes an important point. Incubations are essentially cut off from new C inputs and destabilize soils through homogenization, thereby increasing the potential for older C to be respired. Source is also important – if a stream is draining an older source, it will have an older signal. That is perhaps why the scaling in this paper is so important to the overall interpretation of inland C emissions.

We thank the reviewer for their support on this point. We have also added a concluding sentence to this paragraph in response to Reviewer 2 and to emphasise the main point here (L.241-243).

l. 247. It is also necessary to emphasize here that erosional processes are potentially more important during other times of the year, or during abrupt thaw events. This study occurred only for a short and stable period of the summer, and the proportions of the different C sources cannot be assumed constant throughout the year.

We have added text to this paragraph to include this point (L.284-288).

Figure 1. Adding the ranges for δ13C sources/pathways described in lines 107-121 to the x-axis, similar to how 14C sources are provided on the y-axes, would be informative for interpreting the graph.

Potential $\delta^{13}C$ source signatures are now indicated at the top of this figure – now Fig. 2 (L.318-322).

Figure 2. How many data points were included here? Can you include n values for each bar? It looks like there are far more data points than indicated in Figure 3.

The data points in Fig. 2 (now Fig. 3) represent possible contributions from different sources for the ^{14}C data. The actual ^{14}C datapoints this analysis is based on are shown in Fig. 1 (now Fig. 2). We have included *n*-values in the new Fig. 3.

Table 1. I'm not familiar with the superscript (+) and subscript (-) in the proportion derived from

contemporary sources column. Does that refer to positive and negative uncertainty being different in magnitude?

This is because these values are proportions, and thus can't be greater than 1.00 when the uncertainties are accounted for. So, for example 0.96 ± 0.21 becomes $0.96^{+0.04}_{-0.21}$.

Supporting info:
The data tables were not provided in the review material and could not be assessed.

These data tables were supplied with the submission, so a technical error must have prevented the reviewer from accessing them. These tables contain all the inland water C data collected in this study and are provided for transparency, but are not vital to the interpretation of the manuscript.

--

Reviewer #2 (Remarks to the Author):

Review of: Arctic inland waters emit mostly contemporary carbon

Synopsis:

The authors present a unique and in-depth geochemical analysis (focusing on carbon isotopes) of the four primary carbon pools that effect greenhouse gas emissions from inland waters in a 16km2 study region in north eastern Siberia. The authors show that on the scale of the whole study region, contemporary (i.e. young) carbon sources play a predominant role over ancient permafrost carbon sources in fueling POC, DOC, CH4, CO2 emissions from a variety of inland water types. Using an eddy covariance approach, the authors show that despite high concentrations of dissolved carbon species in the aquatic system, the whole study region was a strong sink of atmospheric CO2. Given this, and the isotopic evidence, the authors conclude that inland waters in permafrost regions (including those with active thermokarst) are mostly regulated by contemporary carbon turnover compared to isolated locations of ancient permafrost carbon release. The study is unique in the sense that it targets dissolved carbon pools which, especially for CH4, are more spatially and temporally integrated than ebullition sources. Furthermore, it provides critical insight into an understudied environment that is undergoing rapid change in response to Arctic amplification. Given the uniqueness of the data, I recommend acceptance of the manuscript, but not before several critical issues are addressed. Please find my general comments and line-by-line comments below.

We thank the reviewer for their positive comments. Their detailed comments are very much appreciated and we feel they have strengthened the manuscript.

General Comments:

• Title is too broad given the limited scope of the study region. In its current form, the title describes the whole Arctic, yet there are individual Arctic lakes that are larger than the defined study area. I would suggest something slightly more representative of the scope of the study. For example: "Inland waters of a Siberian watershed emit mostly contemporary carbon"

We have opted for an option between the reviewer's suggestion and our original title: "East Siberian Arctic inland waters emit mostly contemporary carbon". We feel this captures the spatial scale of the study appropriately in a way that will interest the broad readership of this journal.

• The introduction has too few details about the study site, methods, and approach. More detail is found later on in the methods section; however, I think it would be helpful to for the reader to have more context about the study before jumping in to the results. It would also be helpful if Figure S1 was brought in the main text as Figure 1. Can the probable footprint of the EC tower be added to the figure?

We have added more detailed text to the introduction of the study approach, moving text in from the methods, primarily to explain the sites and their settings (L.81-94). Fig. S1 has been moved to the main text and is now Fig. 1, and the probable footprint of the EC tower has been added to this figure.

• The methods for upscaling EC-tower-based fluxes and assigning relative source proportions to isotopically-defined carbon pools seems somewhat arbitrary. A short straightforward description at lease belongs in the main methods section.

The full methods for calculating and upscaling emissions for the study region, including both for inland waters and eddy covariance tundra fluxes, have been moved into the main methods section from the supplementary materials (L.438-508). We have added a section on the possible carbon source contributions to the different water bodies and how this relates to the isotope mass balance model in the methods (L.407-411), and detail the expected source contributions for the chosen sampling locations in the introduction (L.91-100).

• The authors should emphasize that dissolved CH_4 pools are more spatiotemporally integrated, and thus more representative, than ebullitive CH_4.

A sentence has been added on this into the introduction section on the study approach (L.81-83).

• Some paragraphs required re-reading due to long sentences. Some edits below include suggestions to improve clarity.

The manuscript has been edited to ensure clarity and remove overly long sentences.

Specific Comments:

50 – 52: The certainty of this statement seems to refute lines 33-34 in the abstract that pose this question as a major unknown. No specific recommendation here, but it left me wondering how unknown this topic really is.

Here we have added an additional word to the sentence to clarify: "Inland waters in stable permafrost landscapes primarily receive… [contemporary terrestrial carbon]". What is unknown is the relative importance of contemporary versus pre-aged C contributions to inland waters as these landscapes are impacted by permafrost thaw (see L.65-75).

61: Oxford comma after "…including CO_2)" since CH_4 is organic? Corrected.

78 – 79: Make "a selection of inland water sites" more descriptive and quantitative. See general comment about lack of detail in the introduction.

Text has been added to the introduction to provide more detail early on to the reader on the sampling locations used in this study (L.83-94).

82: What does relatively undisturbed mean? A more detailed description of the site would be helpful. See general comment about detail in the introduction.

We mean where inputs of pre-aged C are limited – this has been added to the text (L.91-94), and more detailed added to this section in general (see our previous responses on this aspect).

86 – 88: I would speculate that very little is known about the seasonality of C emission sources, especially in thermokarst lakes with substantial taliks. One could argue that the oldest emissions of CH4 in thermokarst lakes occur in the fall or even into the winter as the seasonal heat pulse lags through the deeper thawed sediments. See (Elder et al. 2019 JGR: Biogeosciences) which sites evidence of pre-aged CH4 additions to the below-ice dissolved pool occurring throughout the winter season. The authors state that they likely observed the oldest emissions during just the growing season, however this isn't supported by data and is speculation.

This is a good point, and the Elder reference suggested was very helpful in providing further context to the study we present here. In our study, we are specifically referring to the growing season (the sentence reads "… the likely older C released during the growing season…" L.95-96), and for inland waters in general, not just lakes. In our previous work in Canada we showed that the age of inland water carbon does increase over the growing season (Dean et al., 2018 *Environmental Research Letters*) and this mirrors findings in a similar seasonal tundra ^{14}C study (Hicks Pries et al., 2013 *Global Change Biology*), hence our hypothesis that during the summer growing season, we would find the oldest carbon at the end. According to Elder et al 2019, the oldest lake CO_2 and CH_4 across the whole year is likely to be found at the end of winter. We had previously discussed the likelihood that the oldest inland water C across a whole year may be found at the end of winter (L.243-246 in the initial submission). But as the reviewer points out (see Reviewer 2 comment on L.243-246) this was just a logical follow on from that paragraph, rather than a direct finding from our study. So, we now shorten this sentence and moved it to the introduction for context and cite the Elder et al., 2019 paper as evidence (L.97-100).

107: Specify the methanogenic pathway that is implied here, since that matters for the δ13C value found in coexisting CO2. Also, can an actual value replace "high values" here to be more quantitative and informative?

Following this comment and comments from Reviewer 1, we have added in "Potential $\delta^{13}C$ Source Signatures" into Fig. 2. The sentence Reviewer 2 is addressing here has been changed to reflect this (L.122-123). Please note we identify the methanogenic pathway (methyl-type fermentation) later in this paragraph (L.134).

107 – 109: The use of "high values" and "less negative" in these lines is a bit confusing. Does "high" reflect "highly negative?" or high as in very heavy δ13C values? I would suggest choosing a convention and sticking to it (i.e. "very light/very heavy").

The text is corrected to "heavier" or "lighter" for consistency, throughout (L.122-140).

109 – 113: Carbonate sediments/minerals can contribute relatively enriched (heavy CO2) to inland waters. (And 14C-depleted CO2 for that matter) Is there any evidence of carbonate minerals at this site? Should be specified.

Carbonate sediments/minerals do not contribute to the inland waters in this study – this is specified in the site description in the Methods (L.364-368), but is now signposted in this section (L.136-137).

113 – 115: Residual dissolved CH4 pools are also susceptible to enrichment due to partial oxidation/consumption. If thermogenic sources can be ruled out, this is another potential explanation for the relatively heavy δ13CH4 values.

This is a good point, and we have added this possibility to the section here (L. 130-131.

154: Define PARAFAC Corrected (L.174).

193 – 195: This seems like a blanket statement that doesn't hold much meaning. Or is at least a little confusing. Do the authors mean to say that given the predominance of contemporary C in inland waters, these systems are influenced more by the contemporary carbon cycle than the cycling of pre-aged C release? Perhaps some wordsmithing would make this a clearer conclusion.

Yes, that is what we mean, but also that this demonstrates a strong connectivity between these inland waters and contemporary terrestrial C cycling. We have reworded this sentence accordingly (L.216-219).

200: Harsh transition at the beginning of this sentence makes the paragraph hard to follow. Perhaps include "However, the high proportion…" Corrected (L.224).

207: Or that the most labile DOC pools are also the youngest. I think "mobilized carbon" in this sentence applies to all POC/DOC.

We have added in this point to the discussion here. "Mobilized carbon" here is corrected to DOC and POC (L.230-232).

213: At a faster rate or in greater proportion. Not sure if authors can differentiate rates vs. magnitudes of decomposition of the different OC source pools.

In greater proportion – the text is changed to reflect this here (L.238). In this manuscript we are trying to make the point that the shear amount of contemporary C, and the likelihood that it is also very labile, means that contemporary carbon dominates.

213 – 216: Right. Perhaps wrap this idea back around by concluding this paragraph with a statement that young DOC is either decomposed faster or in greater proportion than pre-aged DOC.

We have added a concluding sentence along this line (L.241-243), tying in our response to previous two comments from this reviewer.

235: Reference 37 makes no mention of the 14C age of ebullition CH4. Few studies have compared the 14C age of CH4 in the dissolved pool and ebullition simultaneously (from the same system), However some evidence suggests that 14CH4 ebullition on whole-lake scales can resemble that of the dissolved pool within the same lake, and that a large portion of the dissolved CH4 pool likely originates from ebullition that is trapped and re-dissolved during the ice-cover period. See Elder et al. 2019 Biogeosciences.

Ref. 37 is included for the flux rates, not the age of these emissions. This whole paragraph is about the limitation of contemporary C inputs to inland water systems potentially being the main cause of older CO_2 and CH_4 emissions, rather than the preferential mineralisation of pre-aged C (an important paradigm in recent Yedoma-region literature). This paragraph has been substantially rewritten to

make this clearer, and to draw on the processes highlighted by Elder et al., 2019 (L.258-274).

237: Add semicolon or even a period after “depth.”

Period added, and the start of the next sentence adjusted accordingly (L.264-266).

239 – 240: It seems that seasonal changes in 14C of dissolved CO2 is a very different process than what is being described in this section. Not sure how that is a “same pattern” result, especially since this study did not make seasonal measurements. Either this sentence is out of place, or more description is needed to make that connection.

This section has been rewritten to better show that we use the Alaskan studies of Elder et al. 2018 & 2019 as context for the discussion, and make it clearer that the discussion is about seasonality and emission pathway for both CO2 and CH4 (L.268-274).

242: It is also possible that older, trapped ebullition-CH4 is partially oxidized to CO2 below ice- an “aging” effect on the winter dissolved CO2 pool. Elder et al. 2019

We simplify this sentence (L.269-271) to focus it on the isolation of frozen dissolved CO_2 and CH_4 pools (see previous responses regarding this section).

243 – 246: This is hard to follow- possibly split up into two sentences. See comments above regarding the seasonality of 14C signatures of C emissions. This section is speculation that is not supported by data or reference to other research.

This sentence has been reworded and moved to the introduction for context (L.97-100)

258 – 259: See comment about de-scoping the title of the paper to more appropriately describe the scope of the study region.

We have updated the title as mentioned previously. This paragraph is to acknowledge the limitations inherent in any site-specific study. But given the challenges involved in collecting samples from such remote Arctic field locations, this is a novel and valuable dataset/analysis as pointed out by both reviewers.

265 – 266: But contemporary carbon seems to be the predominant player.

We have added this clause to the sentence for clarity (L.297-298).

347 – 350: Where were lake and pond samples taken? Edges of the water features or from the shoreline? Depending on the heterogeneity of the lake/pond environment, this can have implications for carbon source variability. This should be discussed.

We have added text in the methods to discuss this point and acknowledge the limitations of accessibility and number of analyses (L.383-391)

Supplementary Methods Comments:

46 – 47: Was there a method to check for completeness of combustion? Whether there was or not, this should probably be discussed since there can be significant effects on 14C & 13C results.

This method refers specifically to the measurement of DOC concentration and the independent $\delta^{13}C$-DOC measurements in Leuven, Belgium (separate from the ^{14}C analyses which were analysed at the NERC Radiocarbon Facility in East Kilbride). Samples were analysed using a well described method (e.g. Bouillon et al. 2006, *Limnology and Oceanography: Methods*, doi: 10.4319/lom.2006.4.216; St-Jean 2003, *Rapid Communications in Mass Spectrometry*, doi: 10.1002/rcm.926). These references are now added to the Supplementary Methods (L.742) and a note added to the sentence on calibration regarding the checks for completeness of combustion (L.746).

Checks for completeness of oxidation were confirmed using standards (see example graph below):

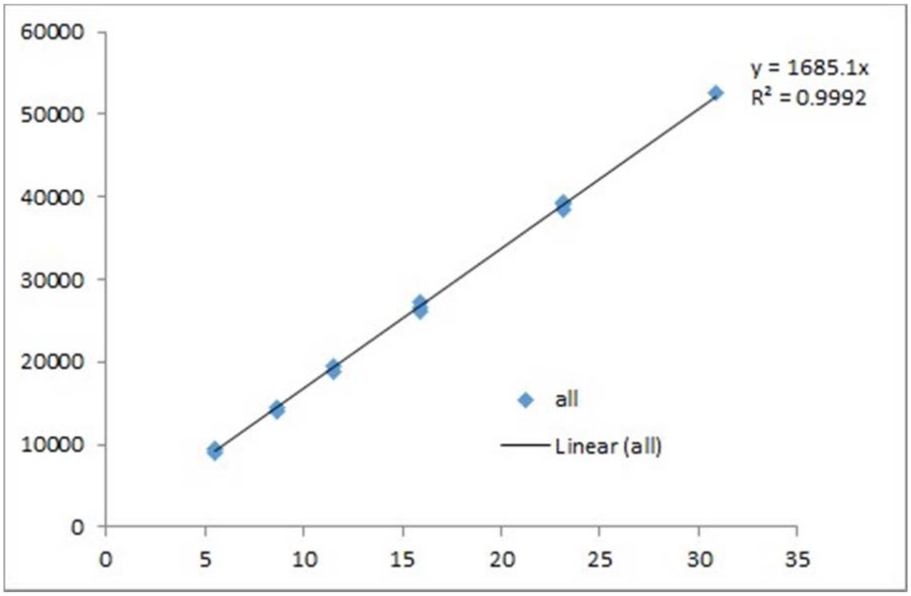

Calibration figure: µg C in standards versus peak area after blank correction.

Concurrent DOC ^{14}C and ^{13}C samples (independent from the Leuven analyses) were processed at the NERC Radiocarbon Lab in East Kilbride to CO_2 by rotary evaporation, acid fumigation, then combusted using standard methods (elemental analyser); radiocarbon standards were processed alongside the samples to verify the ^{14}C and ^{13}C measurements.

160: What is the precision of the 13C measurements from the AMS?

Following consultation with the ^{14}C lab at the University of California Irvine where the small $^{14}CH_4$ samples were processed, the AMS $\delta^{13}CH_4$ values have been removed from the manuscript. These values were not used in the analyses apart from Fig. S8, and removing them has not changed the comparison we make here between $\delta^{13}CH_4$ values samples transferred to gas-tight exetainers in the field or those transported in foil gas bags. The text in the supplement has been corrected appropriately (L.854-855).

179: Is "subsidized" the right word here? Changed to "…supplemented with atmospheric concentration data from the eddy covariance tower." (L.447-448).

222: The selection of the upscaled flux region seems arbitrarily chosen to encompass discrete sampling sites. Should it be determined with footprint analysis instead? What is the footprint of the EC tower? This could have drastic implications for the relative proportions of carbon sources assigned to different flux areas.

Please see our response to this reviewer's general comment on upscaling. The upscaled area was selected to encompass all the sample locations (L.491). This was done so that we captured the direct area to which we knew these measurements applied, but we did not stretch the upscaling beyond this. The footprint of the eddy covariance tower is only a small component of the study area but captures a representative area of tundra that reflects what constitutes the vast majority of the land surrounding the studied inland waters (Fig. 1). The tower does not have any substantial inland water components within its footprint, and the tower is considered to be representative of tundra CO_2 and CH_4 cycling in the region (see e.g. Parmentier et al. 2011 *JGR:Biogeosciences*; doi: 10.1029/2010JG001637). We have added a section on this in the upscaling methods (L.492-497).

References:

Elder, C. D., M. Schweiger, B. Lam, E. D. Crook, X. Xu, J. Walker, K. M. Walter Anthony, and C. I. Czimczik. "Seasonal Sources of Whole-Lake CH4 and CO2 Emissions From Interior Alaskan Thermokarst Lakes." Journal of Geophysical Research: Biogeosciences (2019).

REVIEWERS' COMMENTS:

Reviewer #1 (Remarks to the Author):

The authors have sufficiently addressed the initial reviews and the paper is suitable for publication.

I have two additional requests:

1) change wording from “lighter” and “heavier” to “more negative” and “less negative” when referring to $\delta^{13}C$ ratios. This should clear up the response to the previous reviewer as well. Also make sure to stay consistent throughout the manuscript when using these terms.

2) Please explain what would limit availability of contemporary C in certain systems (l. 240). That is, how are those systems different that the system you study here?

Reviewer #2 (Remarks to the Author):

The authors have done a fine job revising the manuscript. In my assessment, no further revisions are needed other than a minor suggestion to improve the clarity of one sentence:

Line 242-243: move "at the landscape-scale" behind "thawed pre-aged C"

Please find below our responses to the reviewer comments in **blue text**. Corrections in the tracked-changes manuscript file are highlighted yellow. All line references (L.XXX) refer to the tracked-changes manuscript file.

--

Reviewer #1 (Remarks to the Author):

The authors have sufficiently addressed the initial reviews and the paper is suitable for publication.

I have two additional requests:

1) change wording from “lighter” and “heavier” to “more negative” and “less negative” when referring to δ13C ratios. This should clear up the response to the previous reviewer as well. Also make sure to stay consistent throughout the manuscript when using these terms.

All uses of “light” vs “heavy” when describing isotope ratios have been changed to “more negative” or “less negative” throughout – this occurs only in L.122-140.

2) Please explain what would limit availability of contemporary C in certain systems (l. 240). That is, how are those systems different that the system you study here?

A sentence has been added here to provide more explanation (L.241-243): “This effect is amplified by these studies targeting streams heavily impacted by thermokarst activity, and the incubation approaches used which restrict contemporary inputs entirely.”

--

Reviewer #2 (Remarks to the Author):

The authors have done a fine job revising the manuscript. In my assessment, no further revisions are needed other than a minor suggestion to improve the clarity of one sentence:

Line 242-243: move "at the landscape-scale" behind "thawed pre-aged C"

Corrected – L.245.
